# Dual‐Modal Sensing Skin Adaptive to Daylight, Darkness, and Ultraviolet Light for Simultaneous Full‐Field Deformation Measurement and Mechanoluminescence Responses

**DOI:** 10.1002/advs.202409384

**Published:** 2024-10-24

**Authors:** Suman Timilsina, Cheol Woo Jo, Kwang Ho Lee, Kee‐Sun Sohn, Ji Sik Kim

**Affiliations:** ^1^ KNU Research Institute of Artificial Intelligent Diagnosis Technology of Multi‐scale Organic and Inorganic Structure Kyungpook National University Kyeongbuk 37224 Republic of Korea; ^2^ School of Advanced Science and Technology Convergence Kyungpook National University Kyeongbuk 37224 Republic of Korea; ^3^ Department of Automotive Engineering Kyungpook National University Kyeongbuk 37224 Republic of Korea; ^4^ Nanotechnology and Advanced Materials Engineering Sejong University 209 Neungdong ro Gwangjin‐gu Seoul 143‐747 Republic of Korea; ^5^ School of Nano & Advanced Materials Engineering Kyungpook National University Kyeongbuk 37224 Republic of Korea

**Keywords:** digital image correlation, light‐adaptive skin, mechanoluminescence, structural health monitoring, subsurface crack

## Abstract

Mechanoluminescence (ML) and digital image correlation (DIC) have emerged as promising optical methods to visualize and measure deformation fields. In this study, a dual‐modal sensing skin, called the ML‐DIC skin is introduced, that is capable of emitting ML and facilitating DIC measurements under various lighting conditions, including daylight, night or darkness, and UV irradiation. Four ML‐DIC skins are fabricated with or without carbon nanotubes (CNTs) using a composite powder consisting of SrAl2O4: Eu,Dy (SAO), and acrylic resin, with CNT milling times of 48, 72, and 96 h for three of four skins, respectively. DIC measurements are performed under multiple lighting conditions for measuring photoluminescence, persistence luminescence, and reflection. Uniaxial tension tests demonstrate the superior performance of ML‐DIC skins with CNTs compared with pristine SAO skins, with the skin subjected to 48 h of CNT dispersion exhibiting optimal performance. Further investigations focus on ML emission and DIC measurements near the crack‐tip vicinity of static and propagating cracks as well as on surfaces above subsurface cracks. The integration of ML and DIC techniques offers a versatile approach for comprehensive deformation analysis applicable to diverse environments, with implications for materials science, engineering, and structural health monitoring.

## Graphical abstract

A dual‐modal sensing skin for deformation visualization is developed by integrating mechanoluminescence and digital image correlation techniques. It functions effectively under various lighting conditions, such as daylight, complete darkness, and UV irradiation. The sensor can reveal deformations through distinct ML patterns and strain patterns near cracks, including those that are hidden beneath the surface, ensuring comprehensive monitoring.

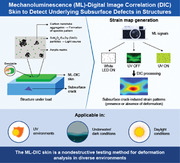

## 1 Introduction

Mechanoluminescence (ML) is a phenomenon in which materials emit light in response to mechanical stimuli such as tension, compression, friction, and fracture.^[^ ^1^, ^2^, ^3^, ^4^, ^5^ ^]^ Given the occurrence of these stimuli across the scales — from single cells in living organisms to large structures such as spacecraft — extensive research has been focused on harnessing ML for advanced stress‐ and strain‐sensing applications in various fields where mechanical forces exist. Applications of ML include structural health monitoring,^[^ ^5^, ^6^, ^7^ ^]^ luminescence anti‐counterfeiting,^[^ ^8^ ^]^ medical health,^[^ ^9^, ^10^, ^11^ ^]^ display,^[^ ^12^, ^13^ ^]^ artificial intelligence skin^[^ ^14^ ^]^ pressure memory,^[^ ^15^, ^16^ ^]^ pressure sensor,^[^ ^17^ ^]^ textile devices,^[^ ^18^, ^19^ ^]^ robot tactile sensing,^[^ ^20^ ^]^ multimode sensing device,^[^ ^21^, ^22^ ^]^ sports science,^[^ ^23^ ^]^ velocity measurement device,^[^ ^24^ ^]^ and other modern intelligent applications in the field of the Internet of Things.^[^ ^25^, ^26^, ^27^, ^28^, ^29^ ^]^ Specifically, stress/strain sensors based on ML materials are used to monitor the proportional relationship between applied stress/strain and ML intensity.^[^ ^30^, ^31^ ^]^

However, translating ML signals into accurate stress/strain measurements remains challenging and is influenced by factors such as ambient light, sensor selection, and calibration methods.^[^ ^3^ ^]^ Despite controlling these factors, ML quantification has been limited to effective stress/strain,^[^ ^5^, ^6^, ^30^ ^]^ as demonstrated in previous experimental studies.^[^ ^5^, ^6^, ^30^ ^]^ Understanding individual strain components, such as axial, transverse, and shear strains, is crucial for comprehending the directionality of deformation in materials with anisotropic properties or defects such as cracks or voids.^[^ ^30^, ^31^ ^]^ This complexity arises because the ML signal results from the collective contribution of strain components, making it difficult to separate the signal into individual strain components. A potential solution to this quantification challenge is to integrate ML with complementary measurement techniques such as digital image correlation (DIC),^[^ ^32^, ^33^, ^34^, ^35^ ^]^ allowing for the simultaneous assessment of ML signals and strain components.

In our recent study, we employed the DIC method to capture full‐field strain components in an ML skin composed of SrAl_2_O_4_:Eu,Dy (SAO) ML particles within an epoxy resin matrix.^[^ ^6^ ^]^ We observed that the SAO particles embedded in the epoxy resin matrix exhibited variations in local intensity under UV exposure, which is a critical characteristic of DIC algorithms. This local‐level inhomogeneous distribution of SAO particles, which results in local intensity variation, is attributed to variations in photoluminescence (PL). By enabling the simultaneous measurement of full‐field ML phenomena and strain components, the coupled ML‐DIC method has been proven to be superior to either the ML or DIC methods alone. Moreover, it allows for the acquisition of both ML and strain information related to full‐field deformation from the same photograph, eliminating the need for separate imaging systems and making measurements both simple and cost‐effective.

Various optical methods, including moiré interferometry, holographic interferometry, fiber optics, caustic methods, and electronic speckle pattern interferometry techniques, have been studied as potential tools for full‐field strain measurement.^[^ ^36^, ^37^ ^]^ However, these methods typically require sophisticated experimental setups and complex specimen preparation techniques, unlike the simpler ML‐DIC method. Recent studies have shown that the DIC method, combined with topology optimization or artificial intelligence, can detect subsurface geometric anomalies, such as cracks and voids in structures, by analyzing defect‐induced patterns in the surface strain field.^[^ ^4^, ^33^ ^]^ Similarly, other studies have observed patterns in the ML field in response to the underlying subsurface defects in the structure.^[^ ^7^, ^31^ ^]^ Consequently, the integrated ML‐DIC method can be more effective in identifying the location and geometry of internal defects in structures by leveraging the advantages of both techniques. This suggests significant potential for ML‐DIC to replace traditional nondestructive testing (NDT) methods, such as eddy current testing,^[^ ^38^ ^]^ ultrasonic testing,^[^ ^39^ ^]^ and X‐ray inspection,^[^ ^40^ ^]^ which often involve expensive sample preparation and complex data acquisition systems and are limited to small‐area inspections without providing strain information. Therefore, considering the potential of the ML‐DIC method as an important tool in structural health monitoring and laboratory mechanical characterization of materials by direct visualization of deformation via ML signals and algorithm‐based strain information, the advancement of the ML‐DIC method is important.

Our previous study on the ML‐DIC method was conducted under UV irradiation,^[^ ^6^ ^]^ which is only applicable to a special class of materials requiring mechanical deformation measurements under UV light or in environments such as outer space.^[^ ^41^ ^]^ However, from a practical application perspective, minimizing UV irradiation is crucial because of the potential health risks, including cancer, making the use of ML‐DIC with UV irradiation in public structures, such as buildings, bridges, aerospace, and ships potentially hazardous.^[^ ^42^ ^]^ Thus, we realized the necessity of upgrading the ML‐DIC method to make it more user‐friendly and environmentally friendly. To satisfy this demand, the ML‐DIC method should be executed under human‐friendly light sources such as daylight or white light from the light‐emitting diode (LED). Furthermore, in some applications, such as underwater structures, the availability of daylight may be minimal or nonexistent. Considering these real‐world scenarios, it is necessary to advance the ML‐DIC method to ensure its functionality in daylight and complete darkness, in addition to UV environments, to enhance its applicability and safety.

**Figure 1:**
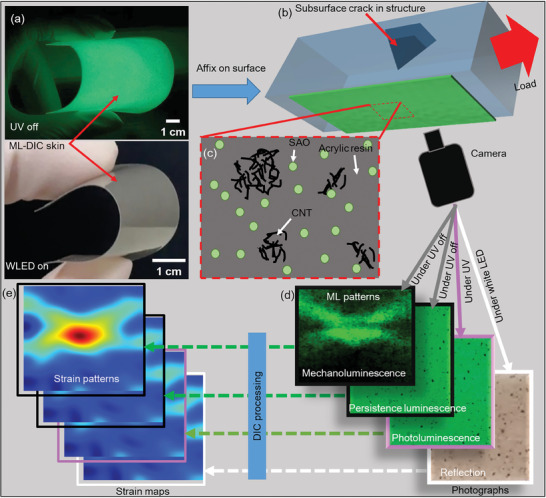
Schematic representation of the multifunctional ML‐DIC skin, highlighting its capability for strain measurements under various lighting conditions and concurrent measurement of ML patterns. a) ML‐DIC skin is exposed to UV and WLED lighting, demonstrating its flexibility. b) Schematic illustration showing the structure with the skin attached to the measurement surface. c) Schematic of the components of the ML‐DIC skin. d) Four types of photographs of the skin under different lighting conditions. e) Strain map generation for each type of photography using DIC algorithms.

In this study, we designed an advanced ML‐DIC skin comprising SAO and carbon nanotubes (CNT) within an acrylic matrix that can efficiently measure the full‐field strain components under UV, white LED (WLED; with a spectrum similar to daylight), and dark ambient conditions without any external light source (see **Figure** **1**). We leveraged the PL and persistence luminescence (PersL) properties of the SAO to capture the images of the ML‐DIC skin respectively under UV irradiation and in a dark environment without an external light source (see Figure 1d). In addition, the reflection of WLED on the ML‐DIC skin facilitated the formation of photographs (see Figure 1d). Importantly, the CNT speckle patterns on the ML‐DIC skin were available under each lighting condition; interestingly, these patterns remained invariant to the ML patterns. Although the SAO acted as a multimodal light source, the CNT contributed to the formation of stable speckle patterns. These synergistic features led to the creation of an advanced ML‐DIC skin capable of conducting DIC measurements under various lighting conditions using a single‐camera system and DIC algorithms, as illustrated in Figure 1e. Therefore, this study offers a versatile method for characterizing deformation fields in daylight and dark environments, as well as in specialized conditions such as UV irradiation. This has significant implications in several fields such as materials science, engineering, and structural health monitoring.

## 2 Results

### 2.1 Grayscale Intensity Distribution in ML‐DIC Skins Under WLED On, UV On, and UV Off Conditions

**Figure 2:**
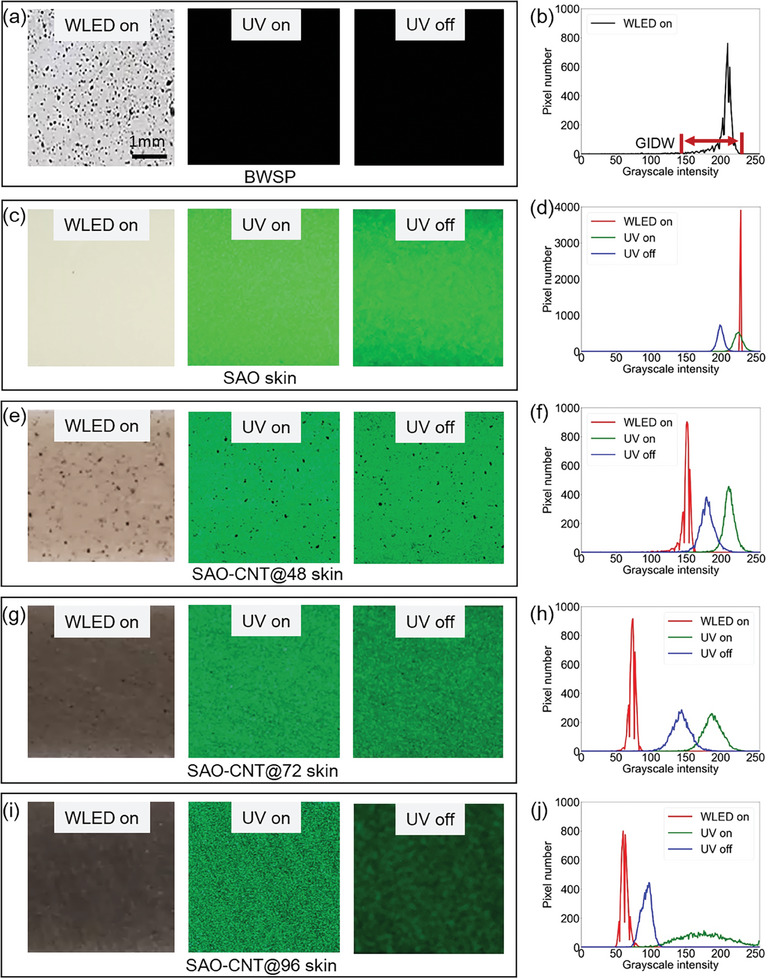
Photographs of BWSP and ML‐DIC skins under WLED on, UV on, and UV off conditions, along with their corresponding grayscale intensity histogram plots. a,b) Photograph and histogram plot of BWSP, which is not visible under UV on and off conditions. Photographs and histogram plots of c,d) the SAO skin, e,f) SAO‐CNT@48 skin, g,h) SAO‐CNT@72 skin, and i,j) SAO‐CNT@96 skin. The histogram plots correspond to regions of 90 × 90 pixels.

The variation in the grayscale intensity distribution on the surface is a crucial characteristic processed by the DIC algorithm to output strain components.^[^ ^35^, ^43^, ^44^, ^45^, ^46^ ^]^ This variation is created by the speckle patterns on the surface. Thus, in the absence of natural texture‐based speckle patterns, artificial patterns are generated to facilitate the DIC measurement. One method for creating artificial speckle patterns on the surface of an object involves spraying white and black paints to form black and white speckle patterns (BWSP), as depicted in **Figure** **2**a.^[^ ^35^, ^43^, ^44^, ^45^, ^46^ ^]^ A standard camera (Sony a7 IV) captured the BWSP under illumination from WLED, as depicted in Figure 2a. The WLED source spectrum (Philips 9290022279) is illustrated in Figure S1a (Supporting Information). Figure 2b illustrates the histogram showing the grayscale intensity distribution of BWSP, determined from a picture subset with a window size of 90 × 90 pixels. The grayscale intensity distribution width (GIDW) was 120 and was derived by subtracting the minimum from the maximum grayscale intensity range, as shown in Figure 2b. It is noteworthy that all experiments were conducted in a dark room, void of sunlight, or regular room light. Because the paints were standard, BWSP was not visible under UV illumination, as shown in Figure 2a. The UV source spectrum (INNO‐CURE 5000) is illustrated in Figure S1a (Supporting Information). BWSP was also not visible in the darkroom, as shown in Figure 2a.

In this study, four ML‐DIC skins with and without CNT incorporation were examined. The fabrication details are described in the Methods section. The ML‐DIC skin shown in Figure 2c, referred to as the SAO skin, was fabricated by heat pressing a powder composite of acrylic resin and SAO (30 wt%) without CNT. These skin‐type mirrors are similar to those reported in our previous research, in which a liquid composite of SAO (30 wt%) and epoxy was directly applied to the surface of the measurement structure.^[^ ^6^ ^]^ Heat pressing was preferred over direct liquid composite deposition because of its precise thickness control and potential for mass sensor skin production. Notably, the performance of SAO skin under WLED on and UV off conditions was not explored in the previous study. Therefore, the performance of the SAO skin was re‐evaluated and compared with that of the CNT‐incorporated ML‐DIC skins in the present study.

Under WLED illumination, the skin exhibited a monotonic texture, whereas, under UV light, it displayed speckle patterns characterized by randomly distributed bright green spots, as shown in Figure 2c. These bright green spots remained observable after the cessation of UV light, as shown in Figure 2c. These spots were the result of PL and PersL from SAO particles, activated under UV light and persisting after the light was turned off, respectively. Both PL and PersL were attributed to 4*f* ^6^5*d* ^1^ → 4*f* ^7^ transitions of *Eu* ^2 +^ ions, resulting in a maximum peak wavelength of 530 nm (green emission).^[^ ^47^, ^48^ ^]^ The PL and PersL spectra are shown in Figure S1b (Supporting Information). The random distribution of SAO particles, which contributes to the speckle pattern under both UV on and off conditions, is evident in the microscopic images shown in Figure S2 (Supporting Information). The random distribution of SAO particles at the local scale is the key to speckle pattern generation. Due to the uneven dispersion of particles, the resulting PL or PersL is emitted non‐uniformly, causing variations in local intensity. These intensity fluctuations create speckle patterns in the photographs. The randomness of particle distribution ensures that no two areas emit light identically, contributing to the distinct nature of the observed patterns. The monotonic texture of the SAO skin under the WLED results from reflection. Although SAO particles emitted PL under WLED irradiation owing to the presence of blue light in the spectrum, the dominant light source captured by the camera was reflective (see Figures S1a,b, Supporting Information). Notably, SAO can be activated by light spectra ranging from UV to blue light.^[^ ^47^, ^48^ ^]^

The monotonic texture of the SAO skin under WLED was further confirmed using a grayscale intensity histogram plot illustrated in Figure 2d. The GIDW was only 7 under WELD, whereas, under UV light on and off conditions, the GIDWs were significantly higher at 50 and 32, respectively. Thus, to enhance the grayscale intensity distribution of the SAO skin under WLED, additional features from the CNT were introduced in this study.

The introduction of CNT was motivated by several factors. The absorbance spectrum of CNT spans from UV to visible light, peaking in the UV range, which is crucial for generating black speckle patterns under all the lighting conditions examined in this study (WLED on, UV on, and UV off).^[^ ^49^ ^]^ Moreover, during mechanical mixing, the CNT aggregates deformed into statistically varied shapes and sizes. Consequently, the color and varied shapes and sizes of the CNT contribute to the generation of speckle patterns that are visible under all the considered lighting conditions. Such diverse speckle patterns from CNT might also help stabilize the pattern of the grayscale intensity distribution when ML patterns are formed, which were verified in this study.

Figure 2e illustrates the CNT‐incorporated ML‐DIC skin, where 0.105 wt% of CNT was added to the SAO skin composition and milled for 48 h. This variation of the skin, owing to its components and mixing duration, is referred to as SAO‐CNT@48 skin. As illustrated in Figure S3a (Supporting Information), the CNT appeared as agglomerates of different morphologies and patches of CNT networks. These patches represent the networks of individual CNT that form after complete deagglomeration and appear less dark than the pristine CNT aggregates. Interestingly, the speckle patterns in Figure 2e closely resemble those of BWSP, as shown in Figure 2a. The corresponding histogram plots in Figure 2f show that the GIDW under WLED on, UV on, and UV off conditions were 70, 97, and 82, respectively, indicating an improvement in the GIDW compared with the SAO skin, especially under WLED illumination. Notably, a 24‐h milling time resulted in uneven distribution, with large CNT aggregates in some regions and a lack of CNT aggregates or networks in others. Therefore, the 24‐h milling process was excluded from this work.

Increasing the milling time to 72 h produced the SAO‐CNT@72 skin, as shown in Figure 2g. This longer milling duration resulted in a further breakdown of the CNT aggregates and an increase in the number of CNT network patches, as illustrated in Figure S3b (Supporting Information). The higher degree of CNT dispersion in the SAO‐CNT@72 skin compared with that in the SAO‐CNT@48 skin led to greater absorption of WLED light, providing the skin with a darker appearance and making only large aggregates visible. Under both UV on and off conditions, the aggregates and patches of the CNT networks were visible. The histogram plots are illustrated in Figure 2h, revealing GIDW values under the WLED on, UV on, and UV off conditions of 42, 85, and 90, respectively.

Furthermore, the SAO‐CNT@96 skin, shown in Figure 2i, was fabricated by extending the milling time to 96 h. At this time, the CNT aggregates were completely deagglomerated, resulting in CNT networks throughout the skin, as illustrated in Figure S3 (Supporting Information). Consequently, the skin was darker under WLED compared with the SAO‐CNT@72 skin. Notably, under UV irradiation, well‐dispersed CNT networks were visible. However, under the UV‐off condition, the contrast decreased as the well‐dispersed CNT networks absorbed more PersL. The histogram plots in Figure 2j show the GIDW values under the WLED on, UV on, and UV off conditions to be 40, 163, and 47, respectively.

In conclusion, the GIDW of the SAO skin under WLED illumination was significantly small, resulting in a monotonic texture. The addition of CNT substantially enhanced the GIDW under both WLED and UV light on and off conditions. This enhancement was observed for all SAO skins with CNT. However, the GIDW of the SAO‐CNT@48 skin under the WLED on, UV on, and UV off conditions was comparatively similar, unlike that of other skins. The correlation between GIDW and measurement accuracy is explored in the following section. Notably, the milling process tends to break down larger CNT aggregates while creating a range of particle sizes, with the overall distribution centered around an average size, forming the bell curve shape characteristic of a Gaussian distribution. The aggregate size distribution remains consistent across all skins when the milling time, rotations per minute (RPM), and mixing container are held constant (see Figure S4, Supporting Information).

### 2.2 Evaluation of DIC Measurement of ML‐DIC Skins with Cyclic Uniaxial Tension Test

**Figure 3:**
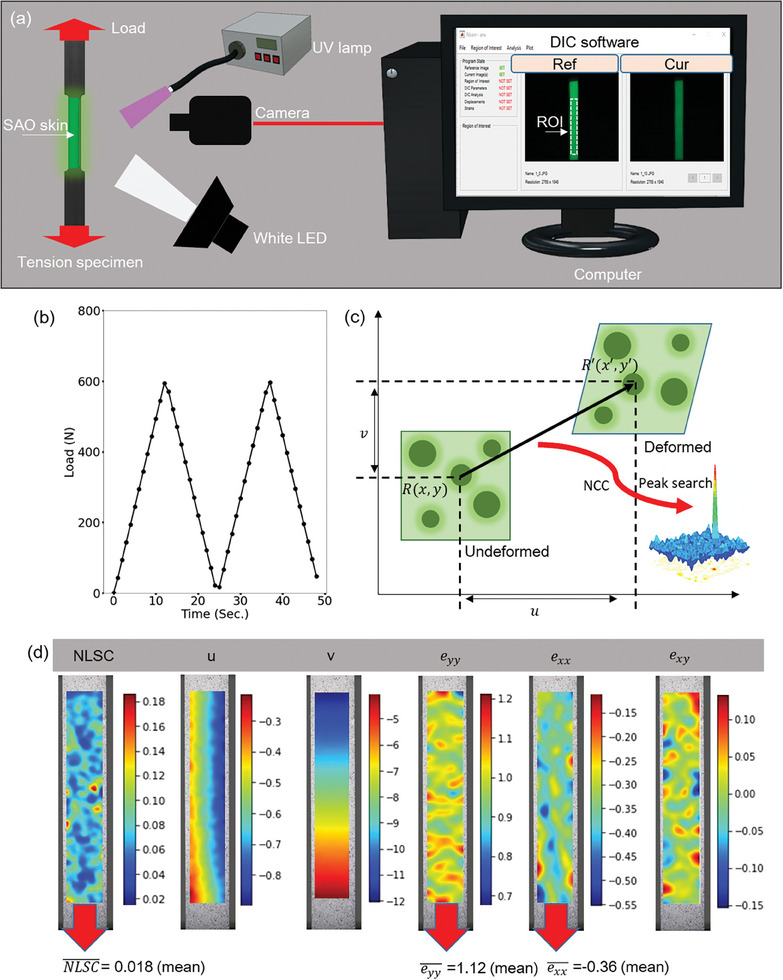
Experimental setup, DIC principle, and possible outcomes from DIC software. a) Schematic illustration of the general setup of the experiment, data acquisition, and DIC software. Experiments are conducted in a darkroom. Subsequent photographs from the camera are fed into DIC software (Ncorr). b) Two cycles of load were applied during the uniaxial tension test with a loading rate of 0.05 mm^−1^s^−1^. c) Schematic diagram of the basic principle of DIC. d) Outcomes from DIC software (Ncorr).

The DIC compatibility of ML‐DIC skins was investigated using an uniaxial tension specimen (UTS) fabricated from an epoxy plate. The UTS dimensions are shown in Figure S5a (Supporting Information). Each ML‐DIC skin was sliced and attached to the gauge section of a different UTS (Figure S5b, Supporting Information). In addition, BWSP was generated on the gauge section of a separate UTS (Figure S5b, Supporting Information), which served as a reference sample for validating the DIC outputs of ML‐DIC skins. The experimental and data acquisition setup is illustrated in **Figure** **3**a. Of note, experiments under UV off conditions were performed 1 s after turning off the UV source, with the UV exposure duration being 3 min. A brief waiting period of 1 s was selected to evaluate the feasibility of DIC measurements during the rapid decay phase of PersL in SAO.^[^ ^3^, ^4^, ^48^ ^]^

Two cycles of load with a peak load of 600 N (see Figure 2b) were applied at a loading rate of 0.05 mm^−1^ s^−1^ using a universal testing machine (E3000). During loading, a standard camera recorded the video at 60 frames per second (fps). Photographs from each experiment (≈48 frames per experiment) were processed using DIC software. In this study, open‐source DIC software written in MATLAB, referred to as Ncorr, was used.^[^ ^32^ ^]^ The same DIC parameters, such as the subset radius (15 pixels), subset spacing (2 pixels), and strain radius (15 pixels), were applied to all measurements.

The two sets of correlation criteria used by Ncorr to determine the location of a deformed subset are briefly described. Initially, the position of the deformed subset is computed as the horizontal displacement (u) and vertical displacement (v) with an integer (pixel) accuracy, which corresponds to the pixel values of the maximum normalized cross‐correlation (NCC) criterion, as illustrated in Figure 3c. The mathematical expression for NCC is provided in Equation 1. The second step employs a nonlinear least‐squares optimizer to refine the results of the first step to subpixel resolution, finding the minimum of a function expressed in Equation 2, which is referred to as the normalized least‐squares criterion (NLSC). An NLSC value close to zero indicates a good match. Furthermore, Ncorr is used to calculate the Green–Lagrangian strains derived from displacement gradients.^[^ ^32^ ^]^

(1) $$\begin{array}{c} NCC=\frac{\sum_{\left(i,j\right)\in S}\left(f\left({\tilde{x}}_{ref_{i}},{\tilde{y}}_{ref_{j}}\right)-f_{m}\right)\left(g\left({\tilde{x}}_{cur_{i}},{\tilde{y}}_{cur_{j}}\right)-g_{m}\right)}{\sqrt{\sum_{\left(i,j\right)\in S}{\left[f\left({\tilde{x}}_{ref_{i}},{\tilde{y}}_{ref_{j}}\right)-f_{m}\right]}^{2}\sum_{\left(i,j\right)\in S}{\left[g\left({\tilde{x}}_{cur_{i}},{\tilde{y}}_{cur_{j}}\right)-g_{m}\right]}^{2}}} \end{array}$$

(2) $$\begin{array}{cc} NLSC & =\sum_{\left(i,j\right)\in S}[\frac{f\left({\tilde{x}}_{ref_{i}},{\tilde{y}}_{ref_{j}}\right)-f_{m}}{\sqrt{\sum_{\left(i,j\right)\in S}{\left[f\left({\tilde{x}}_{ref_{i}},{\tilde{y}}_{ref_{j}}\right)-f_{m}\right]}^{2}}}\\ & {\;-\;\frac{g\left({\tilde{x}}_{cur_{i}},{\tilde{y}}_{cur_{j}}\right)-g_{m}}{\sqrt{\sum_{\left(i,j\right)\in S}{\left[g\left({\tilde{x}}_{cur_{i}},{\tilde{y}}_{cur_{j}}\right)-g_{m}\right]}^{2}}}]}^{2} \end{array}$$

where *f* and *g* are the reference and current image functions, respectively, which return a grayscale value corresponding to the specified (x,y) point; and f_m_ and g_m_ are the mean grayscale values of the final reference and current subsets, respectively.

**Figure 4:**
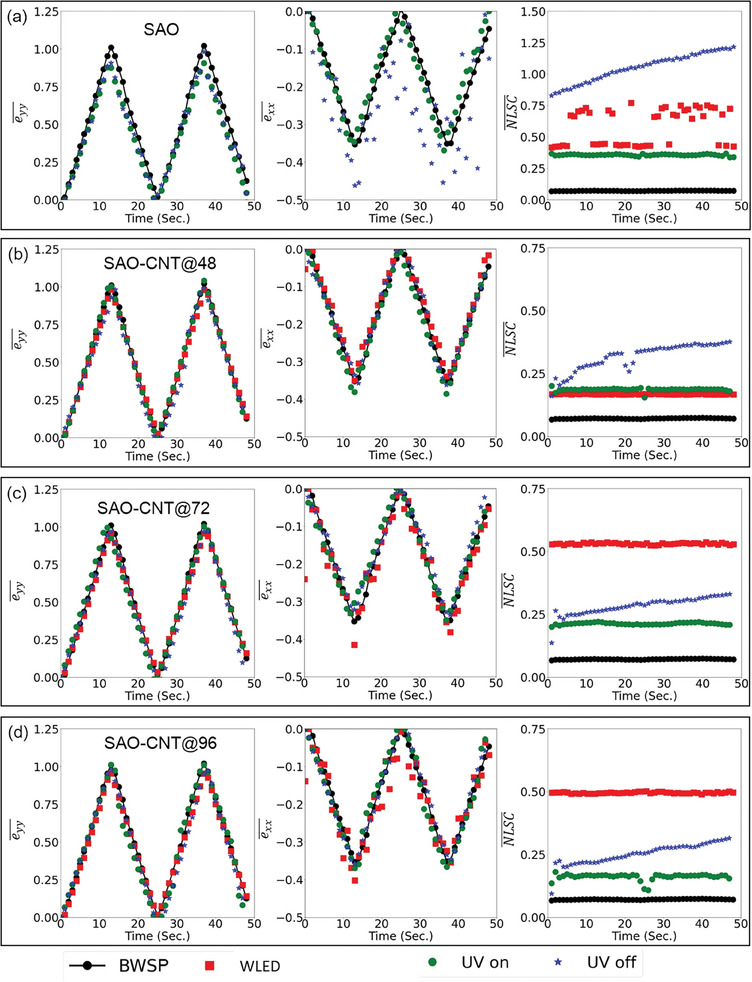
Illustration of the mean longitudinal and mean transverse strain measurements using ML‐skins under various lighting conditions, along with the corresponding mean normalized longitudinal strain components (NLSC). a) SAO skin under UV on and off conditions. b) SAO‐CNT@48 skin under WLED, UV on, and UV off conditions. c) SAO‐CNT@72 skin under WLED, UV on, and UV off conditions. d) SAO‐CNT@96 skin under WLED, UV on, and UV off conditions. Comparative measurements from BWSP are included in the figure. Labels are provided at the bottom of the figure.

The representative outcomes from Ncorr are illustrated in Figure 3d, which were obtained by analyzing a photograph of the BWSP at the peak load (600 N) of the first loading cycle. The strain component maps (longitudinal: *e_yy_*, transverse: *e_xx_*, and shear: *e_xy_*) are expressed as percentage strains. The displacement maps (u and v) are expressed in pixels, whereas the NLSC is a dimensionless parameter. Among the outcomes, *e_yy_* and *e_xx_* were primarily considered when evaluating the performance of ML‐DIC skins because they are the predominant strain components in the UTS. Because they were determined using *u* and *v*, they could sufficiently determine the performance of ML‐DIC skins without evaluating *u* and *v*. The differences between the displacement maps and the strain maps in Figure 3d arise from the use of Green‐Lagrangian strains in Ncorr, which consider nonlinear effects and the full deformation gradient. These strains consider the variations in displacement along both axes, resulting in distinct patterns for strain compared to those for displacement. Moreover, NLSC was examined to understand its influence on strain measurement accuracy. To facilitate a comparison across the two loading cycles, the mean values in each map were computed, as shown in Figure 3d. The results of each experiment are shown in **Figure** **4**.

Figure 4a shows the mean longitudinal strain. $\bar{e_{yy}}$ of the SAO skin under UV on and UV off conditions closely matches that of the BWSP. Although the mean transverse strain $\bar{e_{xx}}$ under the UV on condition and BWSP were similar, $\bar{e_{xx}}$ under the UV‐off condition deviated at several points. Moreover, both $\bar{e_{yy}}$ and $\bar{e_{xx}}$ under the WLED on condition fluctuated significantly, showing considerably higher values than those under BWSP, as shown in Figure S6 (Supporting Information). The $\bar{NLSC}$ of the SAO skin and BWSP are compared and illustrated in Figure 4a. $\bar{NLSC}$ for the BWSP was consistent and approximately equal to 0.018. Similarly, $\bar{NLSC}$ under UV irradiation remained consistent, with a value of ≈0.36. However, $\bar{NLSC}$ under both WLED and UV off conditions was inconsistent, that under the UV off condition continuously decreased, and that under the WLED on condition fluctuated.

The increasing trend of $\bar{NLSC}$ with time under the UV off condition can be attributed to the decaying PersL. The decaying PersL caused the grayscale distribution to shift toward the left as well as a decrease in the GDIW (Figure S7, Supporting Information). Although the subtraction and division of the mean components (*fm* and *gm*) in Equation 2 allowed for $\bar{NLSC}$ to be invariant to shifts in grayscale values and changes in the GIDW, a rapid increase in fluctuation was observed, thus failing to stabilize $\bar{NLSC}$. Consequently, there was a 47% increase in $\bar{NLSC}$ for a 72% decrease in PersL over 48 s. Unlike that under UV‐off conditions, the fluctuation in the mean grayscale intensity was subtle in both BWSP and SAO skins under the UV and WLED irradiation, as shown in Figures S7b–d (Supporting Information). Therefore, shifts in the histogram plots were not observed under these conditions. As a result, $\bar{NLSC}$ remained stable in the BWSP under WLED and SAO skin under UV irradiation. Despite the subtle fluctuation in the mean grayscale intensity under WLED illumination in the SAO skin, the fluctuation in $\bar{NLSC}$ might have stemmed from a small GIDW, which was insufficient for accurate DIC measurements.^[^ ^32^ ^]^

It is important to observe the ML responses of the SAO skin, as depicted in Figure S7 (Supporting Information). The ML responses under UV‐off conditions tended to slow the decay rate of PersL in both regions, as illustrated in Figure S7a (Supporting Information). However, because of the slow loading rate, the ML emissions were too weak to dominate the PersL. ML in SAO demonstrates a dependency on the load and loading rate under UV‐off conditions.^[^ ^1^, ^2^, ^3^, ^4^, ^5^ ^]^ A slow loading rate was intentionally selected to investigate DIC compatibility with PersL, with minimal influence from ML. The feasibility of DIC measurements under significant ML emissions is discussed in Section 2.4. The ML responses were observed under WLED and UV irradiation, as shown in Figures S7b,c (Supporting Information). As mentioned above, the WLED includes blue light to facilitate the charging of traps in SAO. Owing to the continuous recharging of the traps under continuous light exposure, the ML responses were linear and reproducible with load cycles. The ML responses under WLED illumination were noisy, which might be attributed to minor fluctuations in the WLED source, as observed in the BWSP (Figure S7d, Supporting Information). The ML responses under a continuous light source source were reported in our previous studies.^[^ ^5^, ^6^, ^47^ ^]^ Notably, because of the bright background and small magnitude of ML, ML patterns were not observed under continuous light irradiation. Image‐processing algorithms are required to visualize ML patterns.^[^ ^5^, ^6^ ^]^ Conversely, ML responses after turning the UV light off, also referred to as conventional ML, provide high‐contrast ML patterns that are significant for the direct visualization of the deformation field. Therefore, conventional ML was within the scope of this study.

In the SAO‐CNT@48 skin, both $\bar{e_{yy}}$ and $\bar{e_{xx}}$ were consistent with BWSP under all lighting conditions, as depicted in Figure 4b. Importantly, unreliable and unrealistic measurements observed in the SAO skin under WLED illumination were mitigated in the SAO‐CNT@48 skin. Furthermore, $\bar{NLSC}$ of the SAO‐CNT@48 skin, as illustrated in Figure 4b, was significantly lower than that of the SAO skin in each lighting environment. The continuous increase in $\bar{NLSC}$ under UV‐off conditions can be attributed to the decaying PersL, causing a shift in the grayscale intensity distribution and a reduction in GIDW, as shown in Figure S8 (Supporting Information). Moreover, unlike the fluctuating $\bar{NLSC}$ of the SAO skin under WLED illumination, $\bar{NLSC}$ remained consistent in the SAO‐CNT@48 skin.

For SAO‐CNT@72 and SAO‐CNT@96 skins, both $\bar{e_{yy}}$ and $\bar{e_{xx}}$ were consistent with the BWSP under UV on and off conditions. However, when the WLED was turned on, the $\bar{e_{xx}}$ strain component occasionally deviated slightly from the BWSP. The continuous decline in $\bar{NLSC}$ was attributed to the decaying PersL, as shown in Figure S8 (Supporting Information). Furthermore, $\bar{NLSC}$ remained consistent in both SAO‐CNT@72 and SAO‐CNT@96 skins under WLED illumination, although larger $\bar{NLSC}$ values were noted under the WELD on condition than under the UV on and off conditions.

To assess the performance of ML‐DIC skins, the mean absolute errors (MAEs) were computed, as presented in Table S1 (Supporting Information), with BWSP serving as the reference standard. Notably, all ML‐DIC skins incorporating CNT exhibited reduced MAEs under various lighting conditions compared with the SAO skin. For instance, compared with those of the SAO skin, the MAE of $\bar{e_{yy}}$ of the SAO‐CNT@48 skin was reduced by 99.81, 82.57, and 54.63% under WLED on, UV on, and UV off conditions, respectively, whereas the MAE of $\bar{e_{xx}}$ was reduced by 99.81, 65.22, and 84.85%, under these conditions, respectively. Furthermore, the MAE of both $\bar{e_{yy}}$ and $\bar{e_{xx}}$ in the SAO‐CNT@48 skin were comparatively lower than those in SAO‐CNT@72 and SAO‐CNT@96 skins, as presented in Table S1 (Supporting Information). Furthermore, the correlation coefficients between $\bar{NLSC}$, GIDW, and MAEs determined using the data in Table S1 (Supporting Information) are presented in Table S2 (Supporting Information). The negative correlation between $\bar{NLSC}$ and GIDW indicated that $\bar{NLSC}$ tended to decrease with increasing GIDW. A positive correlation between $\bar{NLSC}$ and MAE suggests that measurement accuracy improves with a decrease in $\bar{NLSC}$. Similarly, a negative correlation between GIDW and MAE indicates that the measurement accuracy improves with an increase in GIDW. Therefore, based on the comparatively lower MAEs, a lower $\bar{NLSC}$, and a higher GIDW observed under most lighting conditions, the SAO‐CNT@48 skin was selected as the optimal ML‐DIC skin in this study. Consequently, the SAO‐CNT@48 skin was considered for further experiments to investigate its performance under more challenging conditions, such as edge cracks, subsurface cracks, static and propagating cracks, prominent ML patterns, and higher magnification.

### 2.3 Determination of the Mode I Stress Intensity Factor Under WLED On, UV On, and UV Off Conditions

We showcased the application of ML‐DIC skins in measuring the displacement and strain fields in a UTS under WLED on, UV on, and UV off conditions. Although UTS testing provides crucial material properties, such as yield strength, ultimate tensile strength, and elastic modulus, it may not fully capture the failure behavior of materials in the presence of cracks. Cracks serve as stress/strain concentration zones, quantified by the stress intensity factor (SIF).^[^ ^30^, ^36^ ^]^ Therefore, determining the SIF is essential for assessing the structural integrity of materials and structures. In this context, the optimal ML‐DIC skin (SAO‐CNT@48 skin) was further evaluated to determine the SIF.

In this study, displacement fields were used to calculate the SIF of a crack in an epoxy compact tension shear (CTS) specimen. The dimensions of the CTS and tested CTS specimens are shown in Figure S9 (Supporting Information). To measure the displacement fields, the SAO‐CNT@48 skin, which mimicked the shape of the crack in the CTS specimen, was affixed to one of the surfaces of the specimen (Figure S9b, Supporting Information). Furthermore, a CTS specimen with BWSP was considered for comparative analysis (Figure S9b, Supporting Information). The tests were conducted using a loading cycle with a peak load of 120 N and a loading rate of 0.05 mm^−1^s^−1^. This loading rate was chosen, similar to the tension specimen, to allow DIC measurements with decaying PersL without the influence of ML. Moreover, a peak load of 120 N, which was lower than the fracture load (180 N), ensured that the crack remained static, allowing the same specimen to be used under the WLED on, UV on, and UV off conditions. The deformations were recorded at a frame rate of 60 fps using a standard camera. The relaxation time for the UV off condition was the same as that for the tensile specimen (i.e., 1 s).

**Figure 5:**
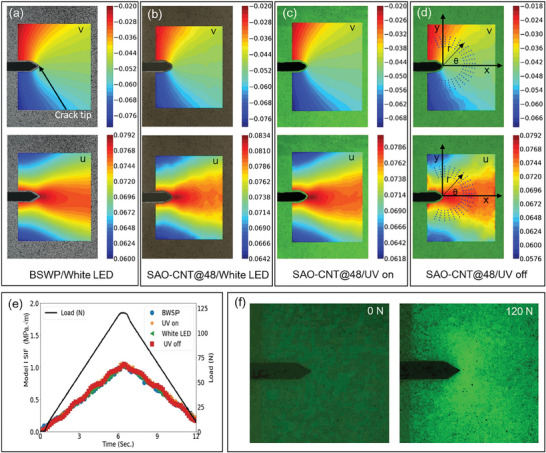
Crack‐tip deformation field and SIF determination under varied lighting conditions. Vertical and horizontal displacement fields at the crack‐tip vicinity of a CTS acquired using a) BWSP, b) SAO‐CNT@48 under WLED on, c) SAO‐CNT@48 under UV on, and d) SAO‐CNT@48 under UV off conditions. e) Determination of the mode I stress intensity factor. f) Visualization of the crack‐tip deformation field through ML.

The displacement and strain fields at the peak load are shown in **Figures** **5**a–d and Figure S10 (Supporting Information), respectively. As shown in Figures 5a–d, both the *u* and *v* displacement fields were smoothly distributed and symmetric relative to the crack axis. This symmetric distribution was attributed to the direction of the applied load being perpendicular to the crack axis, classifying the crack as a mode I crack. Notably, the displacement fields of the SAO‐CNT@48 skin under WLED, UV on, and UV off conditions closely matched those of the BWSP. Similarly, the strain components of the SAO‐CNT@48 skin under these lighting conditions were highly similar to those of the BWSP (Figure S10, Supporting Information).

The displacement fields *u* and *v* near the crack tip are expressed in Equations 3 and 4, respectively,^[^ ^43^ ^]^ where *r* and *θ* represent the distance and angle from the crack tip to a point of interest, as depicted in Figure 5d. Here, *μ* represents the shear modulus, calculated as μ  =  *E*/(2 + 2ϑ) with *E* being the Young's Modulus and ϑ being the Poisson's ratio; *T_x_* and *T_y_* are rigid‐body translation in X and Y directions; R is the rigid body rotation; and X and Y are the coordinates. In Equations (3) and (4), *K_I_*, Tx, Ty, and R are unknown parameters, estimated using a linear least‐squares method^[^ ^43^ ^]^ by considering more than 200 data points from the annular region shown in Figure 5d, which corresponds to 5 mm ≤ r ≤10 mm and −120 ≤ θ ≤120.

(3) $$\begin{array}{c} u=\frac{K_{1}\sqrt{r}}{\sqrt{2\pi }\mu }\;\left[\frac{3-\vartheta }{1+\vartheta }+\frac{1}{2}-\cos\frac{\theta }{2}-\frac{1}{2}\cos\left(\frac{1}{2}-2\right)\theta \right]+T_{x}-\mathrm{RY} \end{array}$$

(4) $$\begin{array}{c} v=\frac{K_{1}\sqrt{r}}{\sqrt{2\pi }\mu }\;\left[\frac{3-\vartheta }{1+\vartheta }-\frac{1}{2}+\sin\frac{\theta }{2}-\frac{1}{2}\sin\left(\frac{1}{2}-2\right)\theta \right]+T_{y}+\mathrm{RX} \end{array}$$

*K_I_* was determined from all sequential photographs recorded during the loading and unloading phases. Figure 5e presents *K_I_* from each experiment, which showed a linear response to the applied load. Importantly, *K_I_* values from each experiment overlapped, indicating that the measurements obtained with the SAO‐CNT@48 skin were both reliable and accurate. Moreover, the MAEs under each lighting condition were calculated using the SIF from the BWSP as a reference. The MAEs were 0.04, 0.03, and 0.02 MPa√m under WLED, UV on, and UV off conditions, respectively, confirming that the errors were minimal. Moreover, *K_I_* values from the SAO‐CNT@48 skin could be accurately measured for loads as low as 10 N. This demonstrates the effectiveness of the SAO‐CNT@48 skin in determining the SIF, even at low levels of deformation.

It is crucial to note the concentration of strain components near the crack tip, as shown in Figure S10 (Supporting Information). With a high strain concentration near the crack tip, ML tended to concentrate in this area. If the load and loading rate are sufficient to trigger ML, the concentration of the ML field at the crack tip can overshadow the PersL background. This phenomenon can be observed in Figure 5f at a loading rate of 0.5 mm^−1^s^−1^, suggesting that ML can significantly aid in the direct visualization of defect sites within a structure. Therefore, exploring the interplay between the conventional ML and DIC algorithms under UV‐off conditions may be particularly insightful.

### 2.4 DIC Measurement with ML Evolution

**Figure 6:**
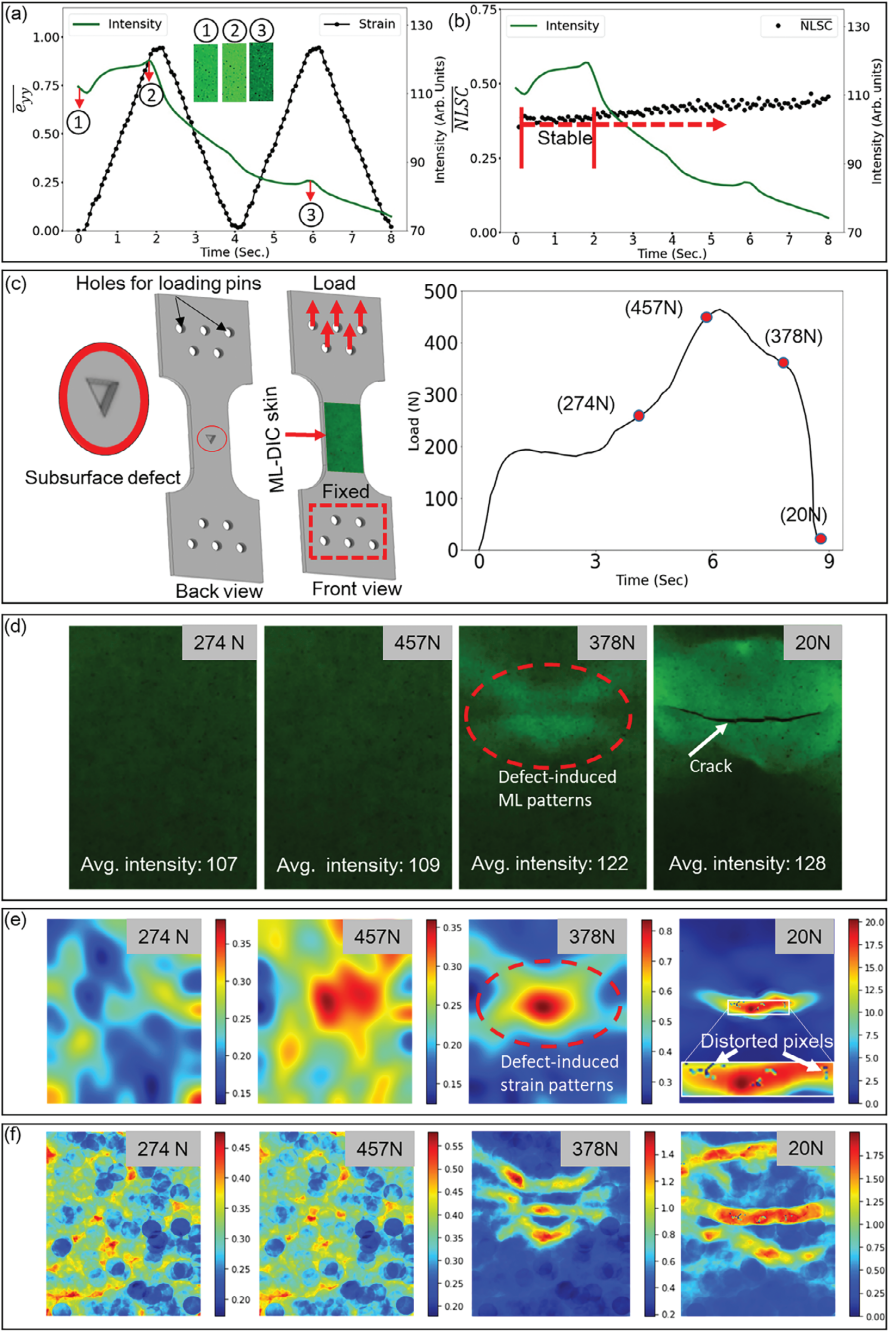
Measurement of strain while ML emission dominates PersL using the SAO‐CNT@48 skin. a) Mean longitudinal strain and average intensity during uniaxial tension. b) The average intensity and the mean NLSC during uniaxial tension. c) Tension specimen with a subsurface defect of triangular geometry alongside the load curve. d) Photographs corresponding to the load levels shown in Figure 6c, displaying von Mises effective strain maps derived from strain components. e) NLSC maps.

**Figure** **6**a illustrates the average intensity and $\bar{e_{yy}}$, determined from the gauge section of the UTS over two loading cycles using the SAO‐CNT@48 skin. The loading rate and relaxation time for PersL were set at 0.5 mm^−1^s^−1^ and 1 s, respectively. The increase in intensity of the ML response during the first loading cycle was more substantial than that during the second cycle. This difference was attributed to the release of the majority of trapped electrons in the conduction band to recombine with the activation center (4*f* ^6^5*d* ^1^ → 4*f* ^7^ transitions of *Eu* ^2 +^ ions) during the first load cycle, thereby producing a significant amount of ML initially.^[^ ^3^, ^47^ ^]^ The histograms of the photographs shown in Figure 6a, as illustrated in Figure S11 (Supporting Information), show rightward and leftward shifts for the second and third photographs, respectively, compared with the first photograph. Despite such dynamic variations in the grayscale intensity, the $\bar{e_{yy}}$ cycles remained relatively stable. Moreover, $\bar{NLSC}$ also appeared stable when the grayscale intensity increased, as illustrated in Figure 6b. This finding is encouraging for future research, paving the way for the use of self‐reproducible ML such as ZnS:Cu to generate mechanically induced speckle patterns for DIC measurements.^[^ ^12^, ^13^, ^14^ ^]^ In contrast to SAO, self‐reproducible ML does not require an external light source for reactivation. Figure 6a illustrates that despite the presence of two peaks of ML due to loading, the PersL decay rate remained relatively stable. This stability arises because ML originates from deeper energy traps, while PersL comes from shallower traps.^[^ ^3^ ^]^ Since both PL and ML contribute to the light used for DIC, their interference does not affect the measurements.

Until now, the tested UTS and CTS specimens were made of polymers. However, it would be beneficial to use metallic specimens to visualize ML and measure the strain. In this context, the UTS was fabricated from aluminum, which is extensively utilized in the aerospace and marine industries. In addition, an artificial defect with a triangular geometry and a depth of 2 mm was introduced into the gauge section, as schematically depicted in Figure 6c. The geometrical dimensions of the defect and the actual specimen used in the tests are shown in Figure S12 (Supporting Information). As the defect did not extend through the thickness of the specimen, it remained invisible from the side to which the SAO‐CNT@48 skin was applied. The ML and strain fields were distributed relatively homogeneously in the UTS, as shown in Figure 6a. Consequently, a subsurface defect was introduced to generate defect‐induced patterns in both ML and strain fields. This approach aimed to demonstrate the feasibility of DIC measurements in conjunction with ML patterns. Furthermore, such demonstrations strengthen the application of the DIC‐ML skin as a nondestructive method for detecting invisible subsurface defects, such as cracks.^[^ ^31^, ^33^, ^34^, ^37^, ^38^, ^39^, ^40^ ^]^

The load curve of the experiment is shown in Figure 6c, with the experimental conditions mirroring those of the UTS, as shown in Figure 6a. Four photographs captured at different load levels (shown in Figure 6c) were considered for further analysis and are illustrated in Figure 6d. At load levels of 274 and 457 N, defect‐induced patterns were not discernible, although there was an increase in the average grayscale intensity in the subsequent images. At a load of 378 N, the defect‐induced patterns in the ML field were visible, approximately mirroring the shape of the subsurface triangular defect. The last photograph, captured at a load of 20 N, reveals the appearance of a surface crack indicating the fracture of the specimen. The gradual drop in load after reaching the maximum suggests that a crack may have been initiated from the subsurface triangular‐shaped defect, but it did not progress to a critical state. The sudden drop in force after 378 N indicates that the crack eventually reached a critical state, leading to the catastrophic failure of the specimen. Figure 6e shows the corresponding effective strain maps. Because ML is widely considered to be triggered by effective strain, these maps highlight the areas of effective strain. The effective strain maps were derived from the strain component maps shown in Figure S13 (Supporting Information) and were calculated using Equation 5:^[^ ^6^ ^]^

(5) $$\begin{array}{c} \mathrm{Effective}\;\mathrm{strain}=\frac{2}{3}\sqrt{e_{xx}^{2}+e_{yy}^{2}+e_{zz}^{2}-e_{xx}e_{yy}-e_{xx}e_{zz}-e_{zz}e_{yy}+3e_{xy}^{2}} \end{array}$$

where ε_*zz*_ was calculated indirectly using Hook's law for the plane stress condition *e_zz_* = ϑ(*e_xx_* +*e_yy_*) with ϑ  =  0.35.

Defect‐induced patterns on the effective strain map were prominently visible at loads of 457 and 378 N. Furthermore, the shapes of the defect‐induced ML and effective strain patterns at 378 N exhibited notable similarities. The strain concentration region at 20 N indicates the presence of a surface crack. Notably, the SAO‐CNT@48 skin could measure up to a 3% strain, aligned with its fracture limit, as shown in Figure S14 (Supporting Information). Values of the effective strain higher than the fracture limit of the skin were observed owing to the crack surface opening. The appearance of distorted pixels at the crack location can be attributed to the loss of skin parts during the fracture. The absence of distorted pixels at 378 and 20 N, except for a few near the crack region, suggests that DIC measurement is possible despite the presence of significant ML patterns. The corresponding NLSC maps are shown in Figure 6f. It is important to note that the higher NLSC values at 378 and 20 N correspond precisely to areas with greater intensity gradients, such as crack openings, resulting in the highest NLSC zone. However, inside the edges of the intensity gradient, the NLSC values remained relatively consistent across all the load levels. Based on the direct correlation observed between the NLSC and measurement error described in Section 2.2, future research should explore methods to minimize the NLSC in regions with high‐intensity gradients. Furthermore, the feasibility of DIC measurements under UV‐off conditions was tested on an aluminum CTS specimen with crack evolution and progression. As illustrated in Video S1 (Supporting Information), the crack advanced by emitting a notable ML. The DIC measurements proceeded smoothly without any notable distorted pixels despite the advancing crack and prominent ML patterns near the crack, as illustrated in Video S2 (Supporting Information).

## 3 Discussion

This study focused on developing advanced ML‐DIC skins that can be used in various lighting environments to measure the strain field on the surfaces of structures. To achieve this, composites of SAO and acrylic resin with and without CNT were used to fabricate four different ML‐DIC skins using the heat‐press method. Among these, three skins contained CNT at the same weight percentage but were milled for 48, 72, and 96 h, respectively. Uniaxial tension tests revealed that the ML‐DIC skins with CNT exhibited more reliable and consistent DIC measurements than those made from pristine SAO under all lighting conditions. Among the developed ML‐DIC skins, the SAO‐CNT@48 skin exhibited optimal performance and was further utilized to measure the asymptotic strain field near the crack tips. Interestingly, the SAO‐CNT@48 skin effectively displayed crack‐tip displacement and strain fields under each tested lighting condition. In addition, the crack‐tip displacement fields were leveraged to determine the SIF, with accurate measurements obtained even at external loads lower than 10 N under all lighting conditions. A notable advantage of ML‐DIC skins is their ability to directly visualize deformation fields through ML emission in addition to performing DIC measurements, as shown in Figure 1. To investigate the potential of DIC measurements with prominent ML emissions compared with background PersL, further tests with an increased loading rate were conducted. These tests confirmed that the strain measurements remained consistent and unaffected by ML emissions. An additional test using a metallic specimen with a subsurface defect also revealed the feasibility of DIC measurements despite significant ML patterns on the measurement surface.

In conclusion, ML‐DIC skins with CNT demonstrated unprecedented performance in DIC measurements under WLED, UV on, and UV off conditions. However, under UV‐off conditions, DIC measurements can be limited to a certain duration owing to the decay of PersL. Theoretically, the maximum duration can exceed 24 h after being fully charged, as PersL in SAO is visible to the naked eye for more than 24 h.^[^ ^50^, ^51^ ^]^ Owing to this long‐lasting PersL, it has been widely used to display emergency signs and indicators during the night or in places where natural light sources are minimal, such as tunnels or under the ocean.^[^ ^52^ ^]^ An advanced imaging system integrated with an intensifier could potentially extend the duration of DIC measurements under dark ambient conditions beyond 24 h; however, this hypothesis must be explored in future studies. It is important to note that charging the ML‐DIC skin is not limited to UV sources. Light sources ranging from blue light to UV rays can be utilized depending on the experimental requirements. Notably, blue light in sunlight can effectively recharge the SAO, ensuring the practicality of ML‐DIC skins for both day and night applications, where sunlight is available for recharging.

The potential for ML‐DIC skins to be utilized in the health monitoring of structures on land, in space, and underwater is promising because of their adaptability to various lighting environments. The skin developed in this study can measure strains below 3%, as demonstrated by the uniaxial tension tests (see Figure S14, Supporting Information). However, several structural alloys can withstand strains over 10% before failure. Hence, to meet this requirement, the skin's stretchability could be increased by adding plasticizers, which would enhance its flexibility and adaptability.^[^ ^6^ ^]^ Notably, the average strain shown in Figures 4 and 6a ML‐DIC skins did not exhibit any significant delay in response during either cycle. This consistency highlights the reliability of the ML‐DIC skins in providing immediate strain feedback, ensuring accurate and timely strain measurements for real‐time structural health monitoring. Furthermore, microscopic imaging of the skin enabled the observation of local deformation at various magnifications (as shown in Figures S15 and S16, Supporting Information) owing to the dispersion of CNT from aggregates to individual particles within the skin. Notably, ML‐DIC skins can be applied to structures with complex geometries owing to the flexibility of the skin, as demonstrated in Figure 1. The flexibility test (see Figure S17, Supporting Information) using a cone demonstrated that the skin maintained its integrity without cracking while being bent around a cone with a calculated radius of ≈1.75 mm. This result indicates that the skin exhibits good flexibility, as it can withstand significant bending without structural failure. Moreover, this method is commercially viable; materials such as CNT, SAO, and acrylic resins are readily available; and the heat‐pressing fabrication method is both simple and cost‐effective, ensuring the scalability and mass production of ML‐DIC skins. Moreover, this method is commercially viable; materials such as CNT, SAO, and acrylic resins are readily available, and the heat‐pressing fabrication method is both simple and cost‐effective, ensuring the scalability and mass production of ML‐DIC skins. Additionally, CNT can be replaced with alternative materials, such as carbon black, which offers a lower cost option compared to CNT. Similarly, SAO can be substituted with other mechanoluminescent materials or PersL phosphors, offering flexibility in material selection based on the application requirements. It is worth mentioning that in our previous study,^[^ ^6^ ^]^ the liquid composite (epoxy + SAO) was applied directly onto the structure in the form of ML paint. However, creating a thin skin with liquid epoxy presents challenges, as the high density of SAO and low density of CNT can lead to distinct layering, adversely affecting the stability of the speckle pattern. Moreover, gas bubbles trapped within the film can weaken the mechanical strength and further disrupt the speckle pattern. Therefore, acrylic resin powder was selected for its easier processing and greater sheet flexibility compared to epoxy skins. Notably, acrylic resin powder remains transparent when heat‐pressed, while most epoxy resin powders suitable for heat‐pressing contain fillers that render them opaque. This transparent matrix is crucial for the transmission of ML photons. In addition to structural monitoring, this technique can be applied to explore the mechanical characteristics of materials. For instance, ML‐DIC skins can be used to investigate trap‐controlled mechanisms in a wide range of ML materials.^[^ ^1^, ^2^, ^3^ ^]^ This is because the ML distribution, displacement fields, and strain fields can all be captured using the same ML‐DIC skin, allowing for a comprehensive study of ML emissions under different loading directions and rates. Such studies are significant for understanding the ML mechanisms across various ML materials. A thorough understanding of the ML mechanism is crucial for synthesizing ML materials with desired multifunctional characteristics.^[^ ^1^, ^2^, ^3^ ^]^ Thus, this study may inspire researchers to explore complex ML mechanisms based on DIC measurements. Dynamic tests, such as fatigue, were not addressed in this study but will be considered in future research. Furthermore, this study examined the mechanical deformation of polymer and aluminum alloy. Future research will explore the application of this method to other materials, such as concrete.

Selecting the appropriate CNT wt% for fabricating DIC‐ML skin is essential. A low CNT wt% results in fewer speckle patterns, which are crucial for tracking strain and displacement in DIC analysis. Insufficient speckle density reduces spatial resolution, making it challenging to detect small or localized changes. Conversely, a high CNT wt% absorbs more ML and PersL, diminishing light emission.

This work conducted a preliminary study using 0.07, 0.105, and 0.14 wt% CNT, while maintaining the SAO content at 30 wt% and the milling time at 48 h to determine the optimal CNT wt%. As shown in Figure S18 (Supporting Information), 0.07 wt% exhibited the fewest CNT aggregates and networks, while 0.14 wt% had the most. Additionally, the transmittance values of 7.7, 6.4, and 5 for 0.07, 0.105, and 0.14 wt% CNT confirm the expected reduction in transmittance with increasing CNT content (Spectro 22RS: 560 nm). Based on these findings, we selected 0.105 wt% CNT for its optimal balance of transparency and CNT distribution, ensuring efficient ML emission and accurate DIC measurements. Notably, all three skins demonstrated similar mechanical performance, as confirmed by the flexibility test using a cone (Figure S17, Supporting Information). Each skin maintained integrity without cracking while being bent around a cone with a radius of ≈1.75 mm, indicating that the differences in CNT wt% were not significant enough to impact mechanical properties.

Optimizing skin thickness is crucial for maximizing ML emission while ensuring DIC measurement accuracy. This study utilized a 100‐micron‐thick skin, consistent with our previous work.^[^ ^6^ ^]^ For accurate DIC measurements and effective strain transfer to SAO particles, the ideal strain transfer ratio between the surface and the skin should be one. Our 100‐micron skin demonstrated excellent strain transfer, with measurements closely aligning with the standard DIC methods. Thinner skins, below 100 microns, may reduce ML intensity due to a lower number of SAO particles. Chen et al. reported that ML intensity increased with thickness up to 245 microns before declining; however, they did not address the strain transfer ratios.^[^ ^53^ ^]^ Hence, our future work proposes to focus on optimizing both ML emission and strain transfer.

## 4 Conclusion

In this study, we fabricated four different ML‐DIC skins to explore the application of DIC algorithms to measure the strain and displacement fields. Three of four skins were produced by heat pressing a composite powder of CNT/SAO/acrylic resin with milling times of 48, 72, and 96 h for the CNT dispersion. A fourth skin was fabricated using the composite powder of SAO/acrylic resin without CNT. The ML‐DIC skin without CNT experienced difficulties in measuring the displacement and strain fields in the gauge section of a uniaxial tension specimen under WLED illumination but demonstrated feasibility under UV on‐and‐off conditions. Conversely, skins with added CNT showed significantly enhanced performance under WLED, UV on, and UF off conditions during uniaxial tension tests. Notably, the skin with 48 h of CNT milling time showed optimal performance, as evidenced by the lower MAEs, lower $\bar{NLSC}$, and higher GIDW across most lighting conditions. Compared with the skin without CNT, the MAE of $\bar{e_{yy}}$ of the optimal skin (i.e., after 48 h of milling) was lowered by 99.81, 82.57, and 54.63% under WLED, UV on, and UV off conditions, respectively. This optimal skin also effectively measured the displacement and strain fields around static and propagating crack tips and detected subsurface defects through patterns in the ML field and effective strain field from the same photographs. Thus, employing ML‐DIC skins to detect and characterize surface and subsurface defects or cracks through ML emission and DIC measurements has heralded the development of sophisticated NDT techniques. Moreover, the feasibility of using ML‐DIC skins under higher magnification was demonstrated, allowing for the multiscale assessment of deformation with enhanced precision and detail. The integration of multimodal light sources from SAO and stable speckle patterns from CNT dispersion facilitates the application of DIC in various environments, potentially ranging from underground to outer space, and is adaptable to varying light ambients. Furthermore, the study's framework can be applied to unravel the complex trap‐controlled ML mechanism, which is crucial for advancing the development of ML materials.

## 5 Exprimental Section

### Materials

SAO (diameter: ≈30 µm) was obtained from Nemoto & Co., Japan. Excitation and emission peaks of SAO were observed at 360 and 530 nm, respectively. Multi‐walled carbon nanotubes (diameter: ≈15 nm, length: ≈10 µm, and purity: ≈90 wt%) were sourced from Carbon Nano‐Material Technology Co., Korea. The epoxy resin and hardener were acquired from Resoltech (USA). Acrylic resin powder, which became optically transparent upon hot pressing, was purchased from Struers ApS (Denmark). The aluminum alloy 6061‐T6 (AA6061‐T6) was procured from a local supplier in South Korea. White and black spray paints were purchased from TOOLSMRO Company. Super glue gel cyanoacrylate (clear) was purchased from the commercial market in South Korea for attaching the skin to the substrate.

### Skin Fabrication

In this study, four types of ML‐DIC skins were produced. One type of ML‐DIC skin was fabricated from a blended powder composite of acrylic resin and SAO (30 wt% of acrylic resin) by heat pressing (Heating Plate Tester, QM900 M). The other three types of ML‐DIC skin were fabricated using a blended powder composite of acrylic resin, SAO (30 wt% of acrylic resin), and CNT (0.105 wt% of acrylic resin), with mixing durations of 48, 72, and 96 h, respectively.

**Figure 7:**
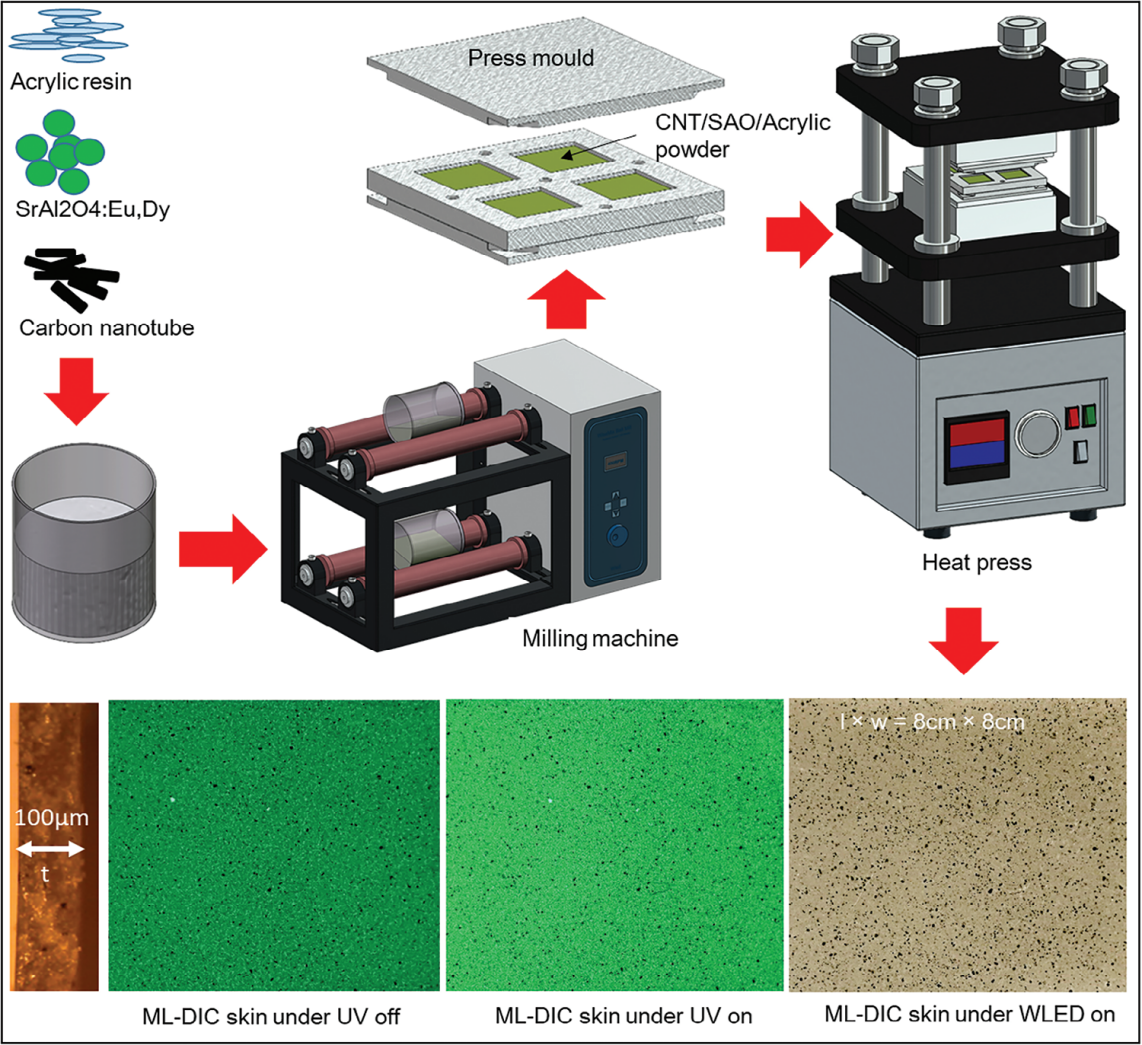
Illustration of the steps involved in preparing ML‐DIC skins.

The mixing process was performed using a milling machine (WiseMix Ball Mill), with the revolutions per minute set to 150. A custom‐designed press mold produced four ML‐DIC skins with dimensions of 8 cm × 8 cm × 100 µm (length × width × depth). The heat press was applied at a pressure of 20 MPa, and the mold temperature was set to 200 °C for 20 min. Following the heating phase, the temperature of the press mold was lowered by circulating cold water. A schematic illustrating the general steps involved in the fabrication of the ML‐DIC skin is shown in **Figure** **7**. The ML‐DIC skins had an area of 8 cm × 8 cm and a thickness (t) of 100 µm.

### Sample Fabrication

Tension and CTS specimens were fabricated from an epoxy plate using a water jet machine. The epoxy plate consisted of a 10:3.5 weight ratio of epoxy resin to hardener. A tension specimen with a triangular geometry defect was fabricated from an aluminum plate using a water jet machine.

### Statistical Analysis

To assess the performance of ML‐DIC skins, the mean absolute errors (MAEs) of average strain were computed, as presented in Table S1 (Supporting Information), with BWSP serving as the reference standard. Furthermore, the correlation criteria between $\bar{NLSC}$, GIDW, MAE $\bar{e_{yy}}$, and MAE $\bar{e_{xx}}$ were determined using the data in Table S1 (Supporting Information) are presented in Table S2 (Supporting Information). Furthermore, the MAEs of SIF under each lighting condition were calculated using the SIF from the BWSP as a reference. Python (version 3.7) was used to calculate the absolute errors and correlation coefficients.

## Conflict of Interest

The authors declare no conflict of interest.

## Supporting information



Supporting Information

Supplemental Video1

Supplemental Video 2

## Data Availability

The data that support the findings of this study are available from the corresponding author upon reasonable request.
