# Supplementary material for: Dual‐Modal Sensing Skin Adaptive to Daylight, Darkness, and Ultraviolet Light for Simultaneous Full‐Field Deformation Measurement and Mechanoluminescence Responses
Source: Adv Sci (Weinh). 2024 Oct 24;12(2):2409384. doi: 10.1002/advs.202409384 (PMC11727239; doi:10.1002/advs.202409384)
Supplement: Supplementary file 1 — Supporting Information [file ADVS-12-2409384-s003.docx]

Supporting Information

**Dual-Modal Sensing Skin Adaptive to Daylight, Darkness, and Ultraviolet Light for Simultaneous Full-Field Deformation Measurement and Mechanoluminescence Responses**

*Suman Timilsina, Cheol Woo Jo, Kwang Ho Lee*, Kee-Sun Sohn* and Ji Sik Kim**

**
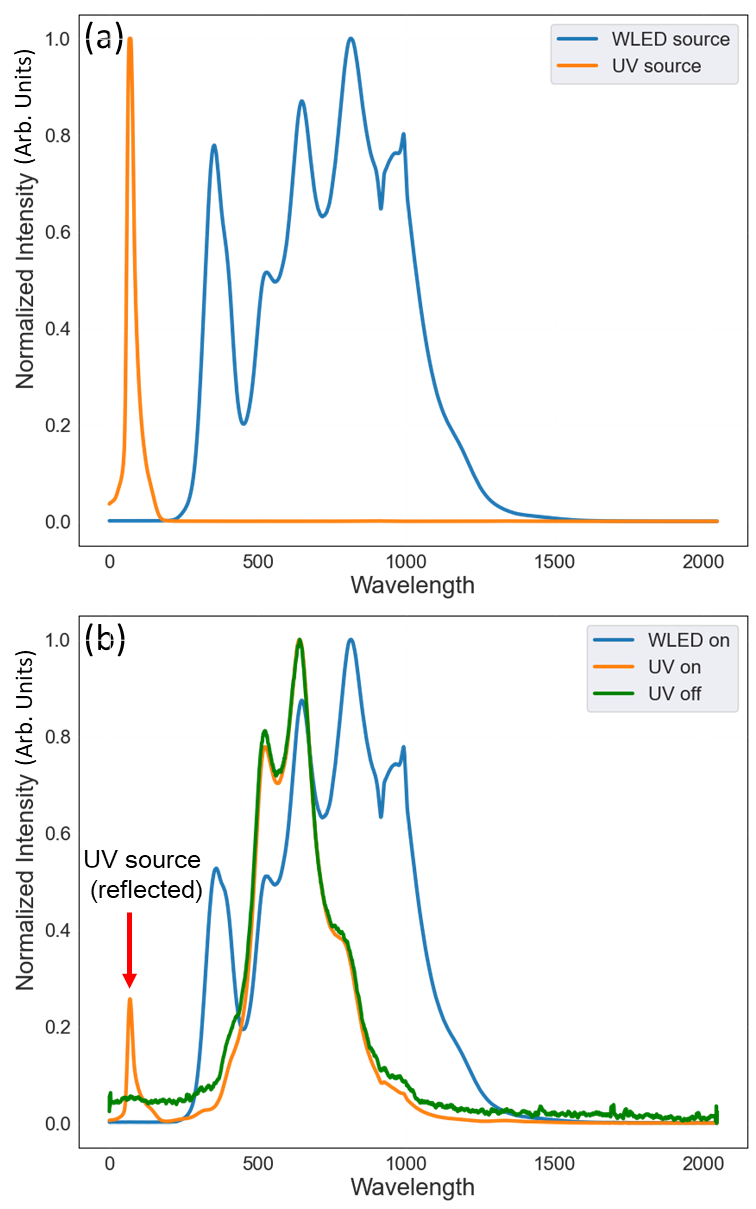
**

**Figure S1.** (a) Normalized emission spectra of the white light-emitting diode (WLED) and ultraviolet (UV) sources. (b) Normalized emission spectra of the SrAl2O4: Eu,Dy (SAO) skin under WLED on, UV on, and UV off conditions. Photoluminescence (PL) is emitted when the UV light is on, whereas persistence luminescence (PersL) is emitted after the UV light is turned off. The spectrum of the skin under the WLED resembled the WLED source spectrum, except for a reduced intensity in the blue light region. The SAO also absorbs blue light, which triggers both PL and PersL.

**
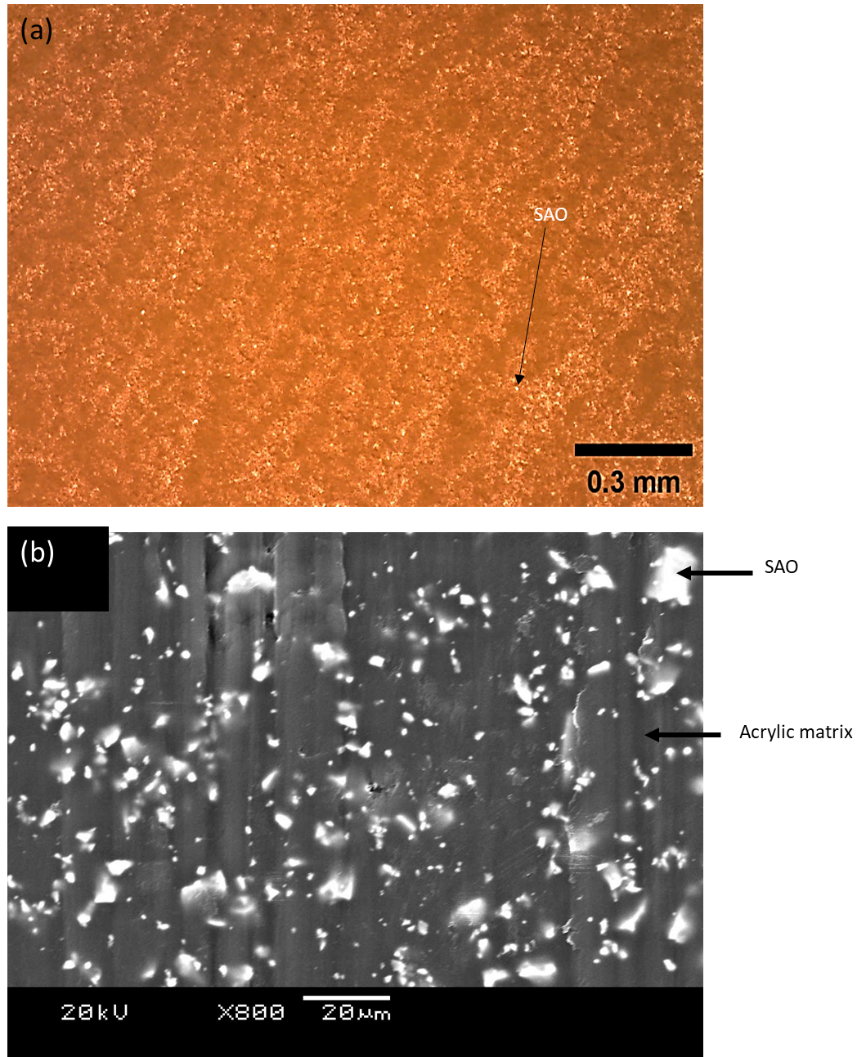
**

**Figure S2.** (a) Optical image of the SAO skin captured using a digital microscope at 250× magnification, showing bright speckles of SAO microparticles. (b) Scanning electron microscopic image of the SAO skin at 800× magnification.


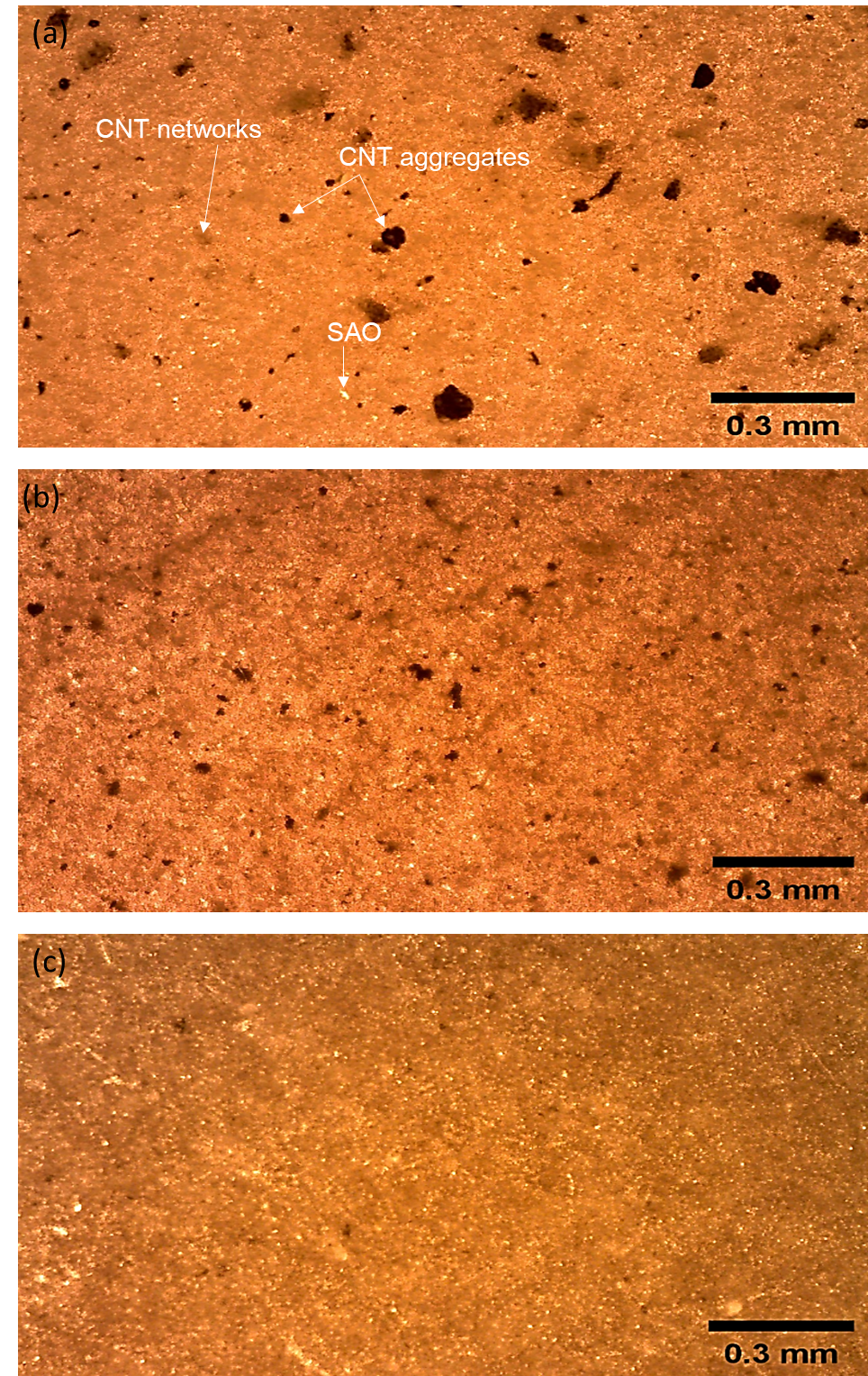


**Figure S3.** Optical images of (a) SAO-CNT@48, (b) SAO-CNT@72, and (c) SAO-CNT@96 skins captured using the digital microscope at 250× magnification. The bright speckles in the images are the SAO microparticles. CNT aggregates appear in (a) and (b), with the size of the aggregates being smaller in (b). As shown in (c), the CNT aggregates are completely deagglomerated into CNT networks.

**
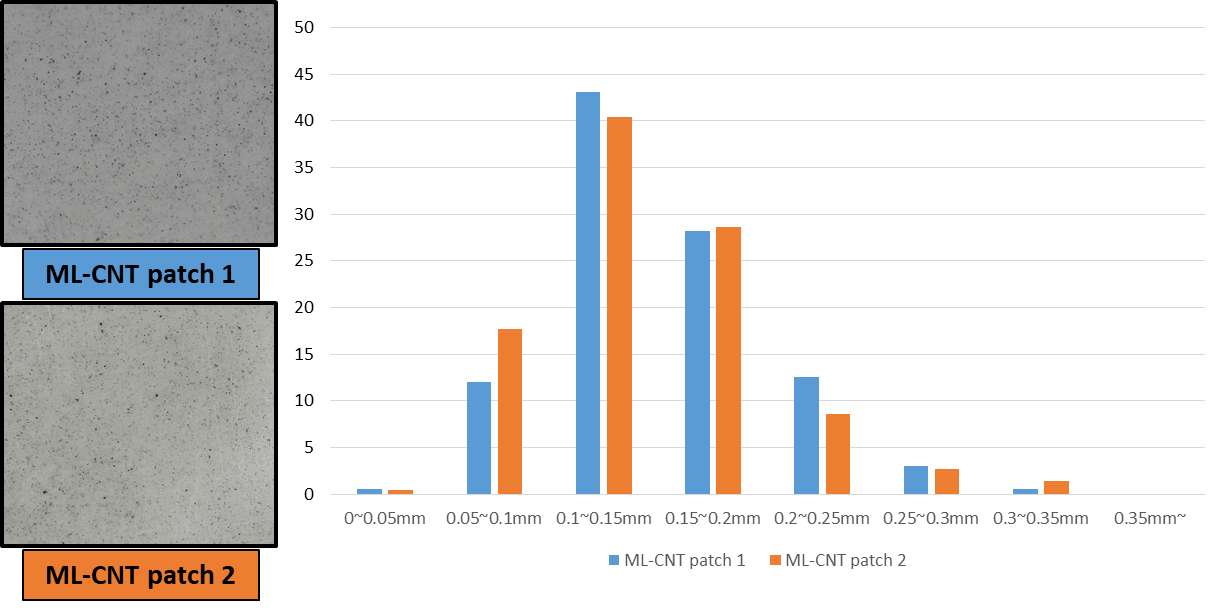
Figure S4.** CNT aggregates size distribution in SAO-CNT@48 skin. The particle size was estimated using ImageJ software.

**
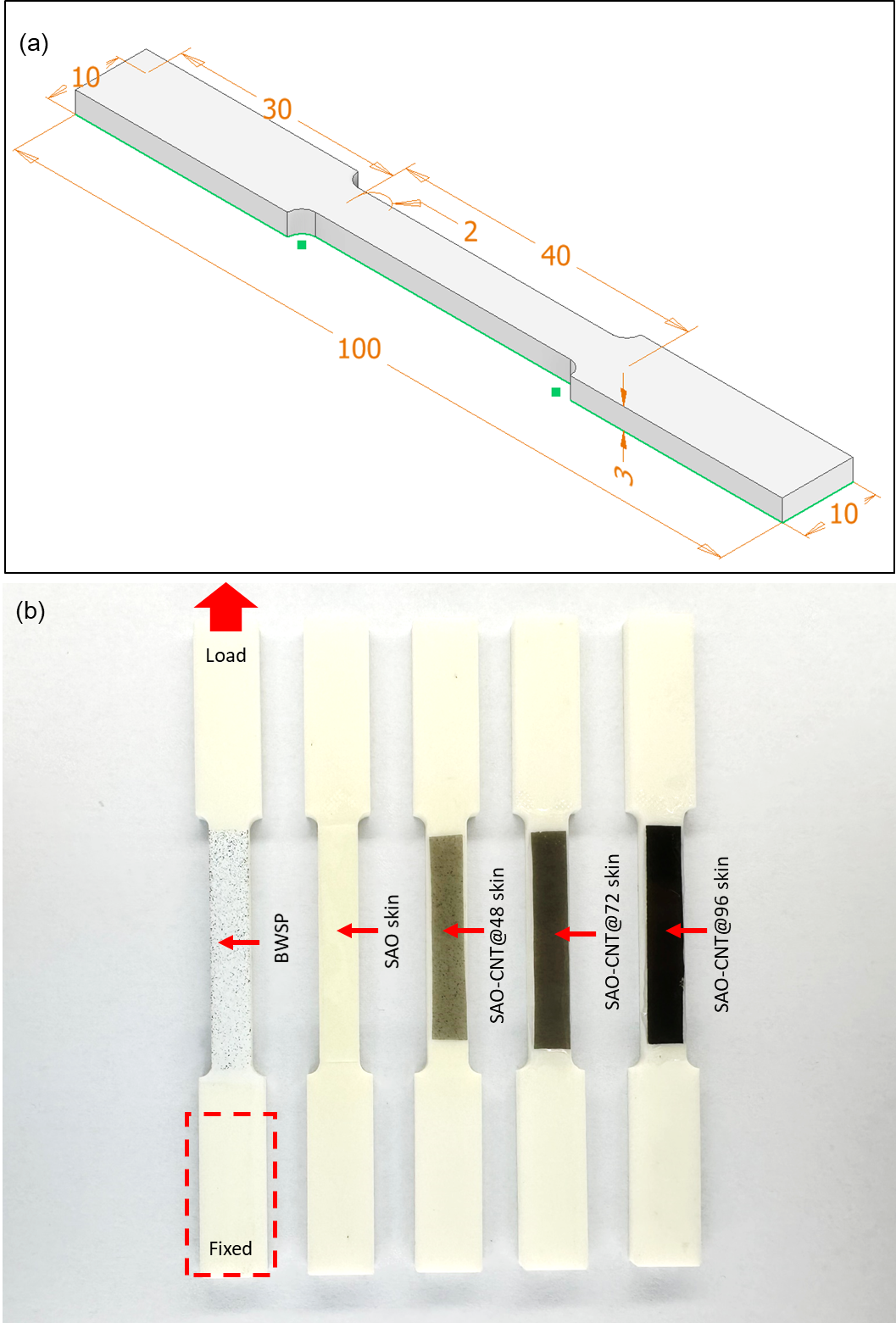
**

**Figure S5.** (a) Schematic illustration of the uniaxial tension specimen with specific dimensions. (b) Uniaxial tension specimens with fixed ML-DIC skins.

**
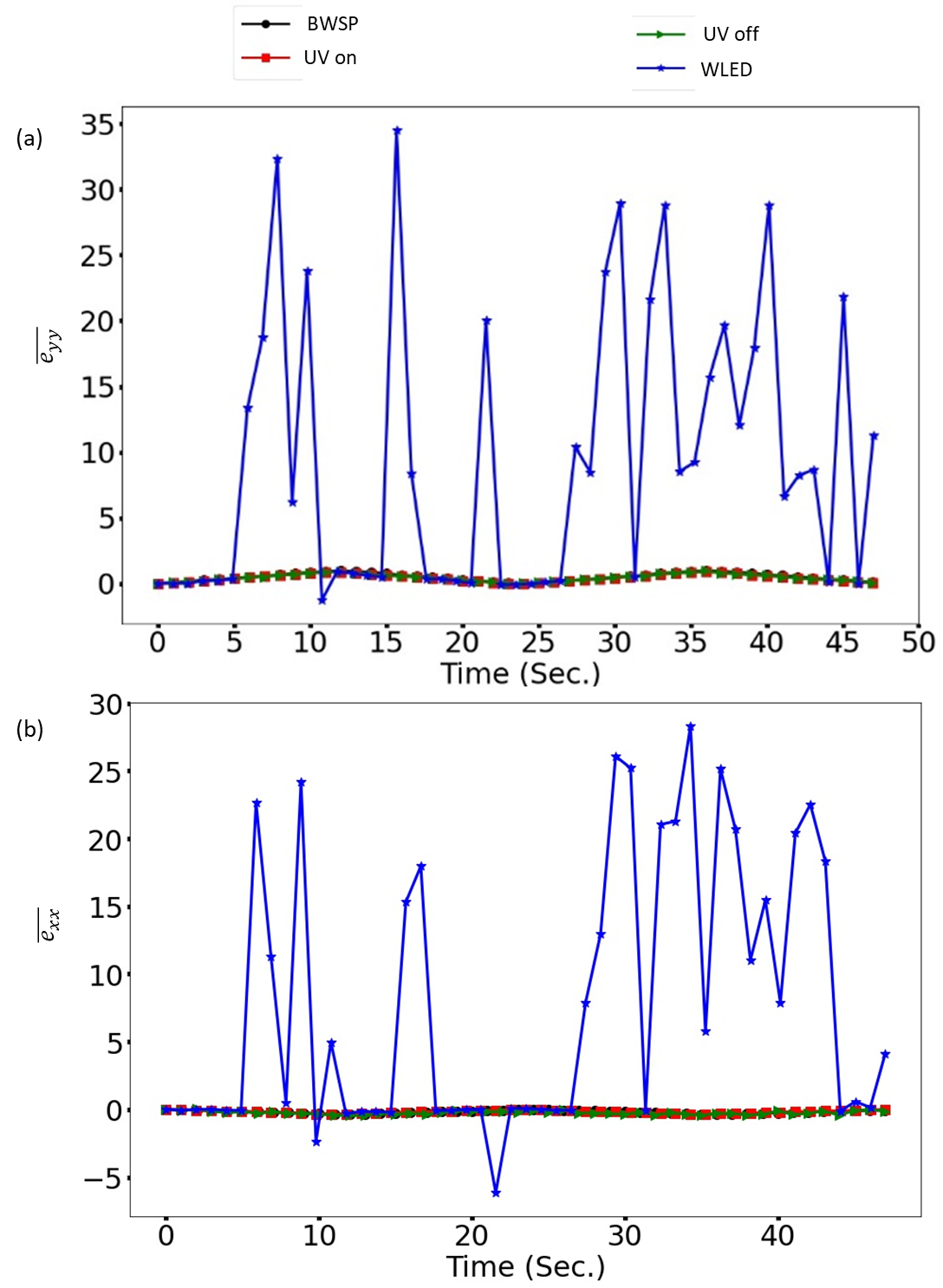
Figure S6.** Illustration of the mean longitudinal strains (a) and mean transverse strains (b) of the SAO skin under WLED, UV on, and UV off conditions compared with those of black and white speckle patterns (BWSP). The measurements under WLED produced unrealistically high strain magnitudes.


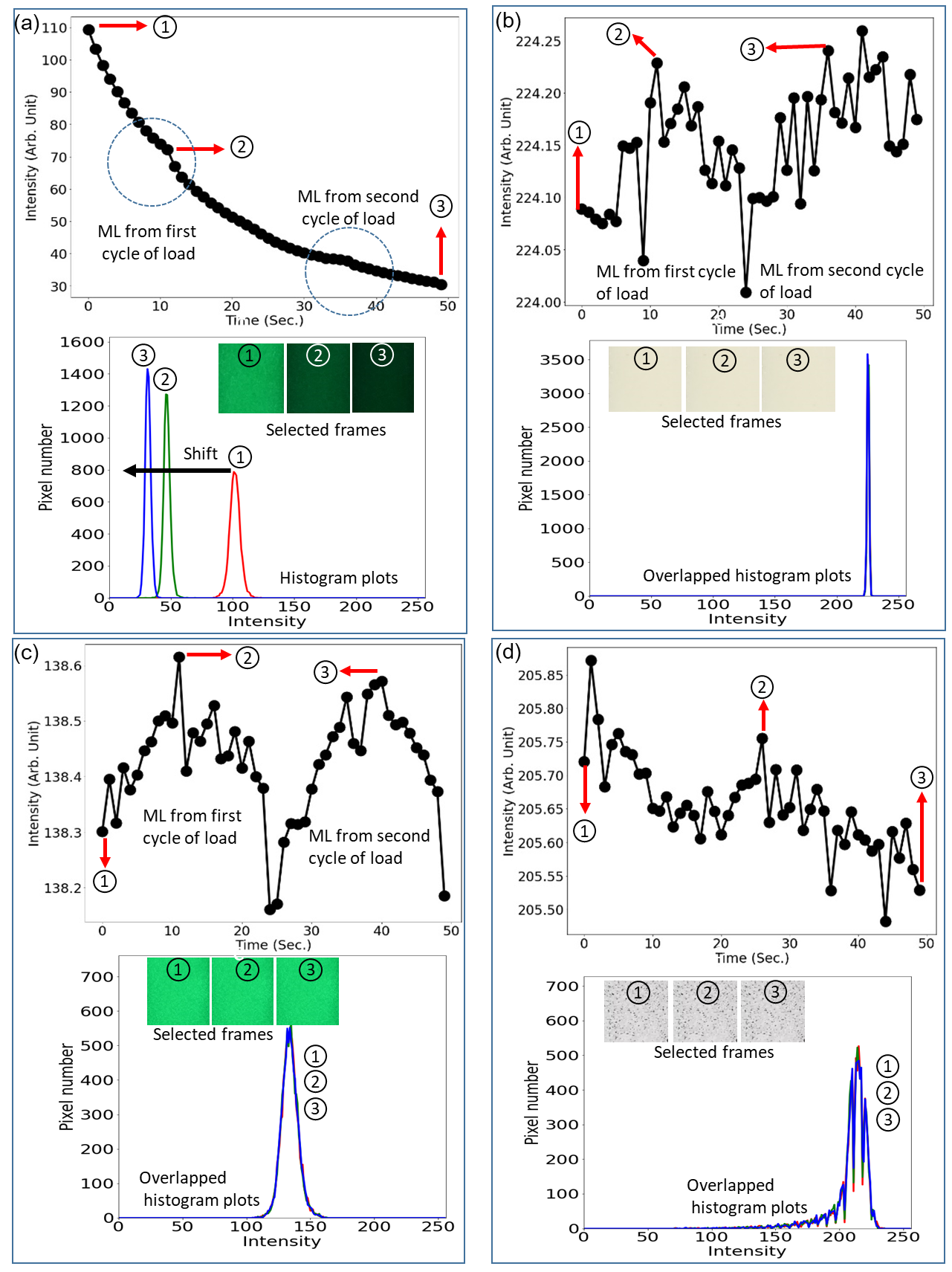


**Figure S7.** Average grayscale intensity and histograms of (a) the SAO skin under UV light, (b) SAO skin under WLED irradiation, and (c) SAO skin under UV irradiation. (d) BWSP under WLED. The average grayscale intensity plots in Figure 4(a) represent the average intensity of each sequential photograph used for DIC measurements. The average intensity was determined from the region of interest (ROI) used for the DIC measurements. The histogram plots correspond to photographs captured at different sequential locations, as indicated by the red arrows in the figures. The histograms overlapped, except in the UV off condition.


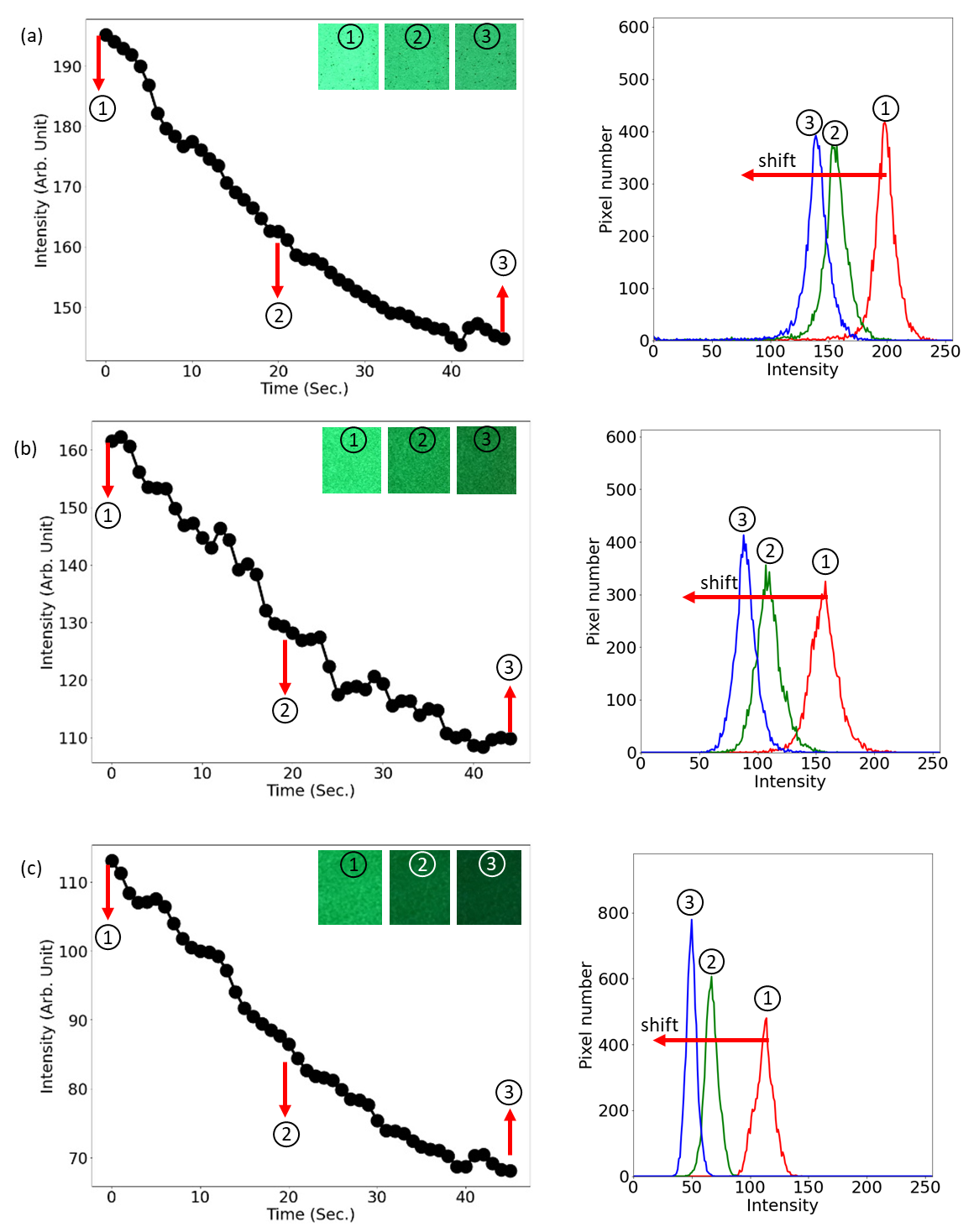


**Figure S8.** Average grayscale intensity and histogram plots of (a) the SAO-CNT@48 skin without UV irradiation, (b) SAO-CNT@72 skin with UV irradiation, and (c) SAO-CNT@96 skin with UV irradiation. The average grayscale intensity plots represent the average intensity of each sequential photograph used for DIC measurements under UV off conditions. The average intensity is determined from the ROI used for the DIC measurements. The histogram plots correspond to photographs captured at different sequential locations, as indicated by the red arrows in the figures.

**Table S1.** Illustration of $\bar{NLSC}, GIDW$, and MAEs of ML-DIC skins and BWSP under various lighting conditions. MAEs and $\bar{NLSC}$ are obtained from the results illustrated in Figures 4 and S5. The MAEs of ML-DIC skins are calculated using BWSP values as a reference. $\bar{NLSC}$ and grayscale intensity distribution width (GIDW) under UV off conditions are expressed as ranges corresponding to the first and last photographs. $\bar{NLSC}$ under WLED of the SAO skin is also listed in the range showing fluctuations.

| Pattern/skin | Light source | $\bar{NLSC}$ | GIDW | MAE $\bar{e_{yy}}$  (% strain) | MAE $\bar{e_{yy}}$  (% strain) |
| --- | --- | --- | --- | --- | --- |
| BWSP | White LED | 0.06 | 120 | Ref. | Ref. |
| SAO | White LED | 0.4–0.78 | 7 | 9.4 | 9.3 |
| SAO | UV on | 0.36 | 50 | 0.109 | 0.046 |
| SAO | UV off | 0.82–1.21 | 32~18 | 0.108 | 0.099 |
| SAO-CNT@48hr | White LED | 0.16 | 70 | 0.018 | 0.018 |
| SAO-CNT@48hr | UV on | 0.18 | 97 | 0.019 | 0.016 |
| SAO-CNT@48hr | UV off | 0.28–0.37 | 82~91 | 0.049 | 0.015 |
| SAO-CNT@72hr | White LED | 0.53 | 42 | 0.024 | 0.018 |
| SAO-CNT@72hr | UV on | 0.2 | 85 | 0.047 | 0.021 |
| SAO-CNT@72hr | UV off | 0.23–0.35 | 90~60 | 0.043 | 0.021 |
| SAO-CNT@96hr | White LED | 0.49 | 40 | 0.029 | 0.027 |
| SAO-CNT@96hr | UV on | 0.16 | 163 | 0.048 | 0.022 |
| SAO-CNT@96hr | UV off | 0.20–0.32 | 47~32 | 0.046 | `0.016 |

**Table S2.** Correlation criteria between $\bar{NLSC}$, GIDW, MAE $\bar{e_{yy}}$, and MAE $\bar{e_{xx}}.$ The correlation criteria range from -1 to 1.

|  | $\bar{NLSC}$ | GIDW | MAE $\bar{e_{yy}}$ | MAE $\bar{e_{xx}}$ |
| --- | --- | --- | --- | --- |
| $\bar{NLSC}$ | 1.00 | -0.73 | 0.71 | 0.89 |
| GIDW | -0.73 | 1.00 | -0.59 | -0.63 |
| MAE $\bar{e_{yy}}$ | 0.71 | -0.59 | 1.00 | 0.86 |
| MAE $\bar{e_{xx}}$ | 0.89 | -0.63 | 0.86 | 1.00 |


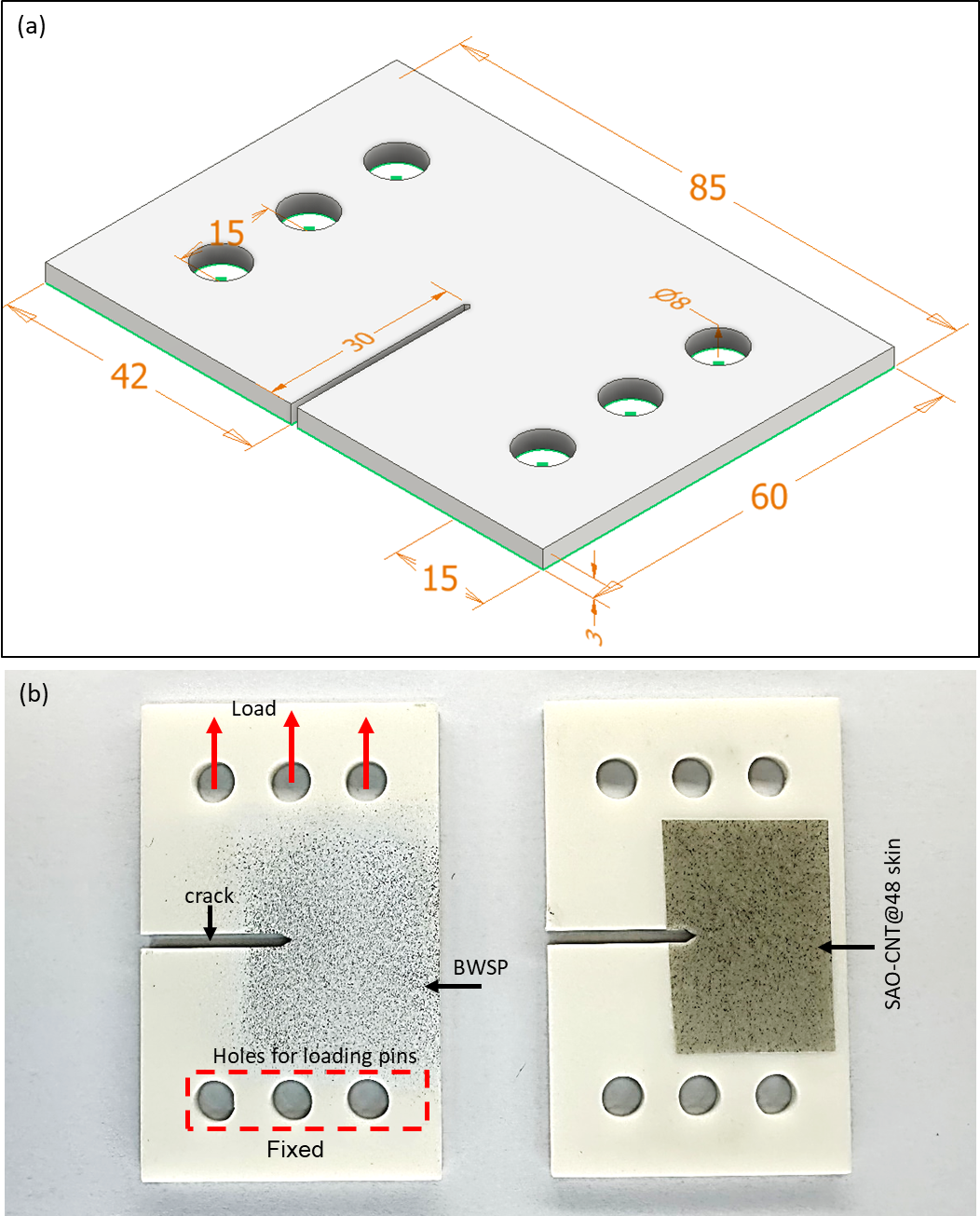


**Figure S9.** (a) Schematic illustration of the compact tension specimen with dimensions. (b) Actual compact tension specimens fabricated from epoxy plates with the BWSP (left) and SAO-CNT@48 skins (right).


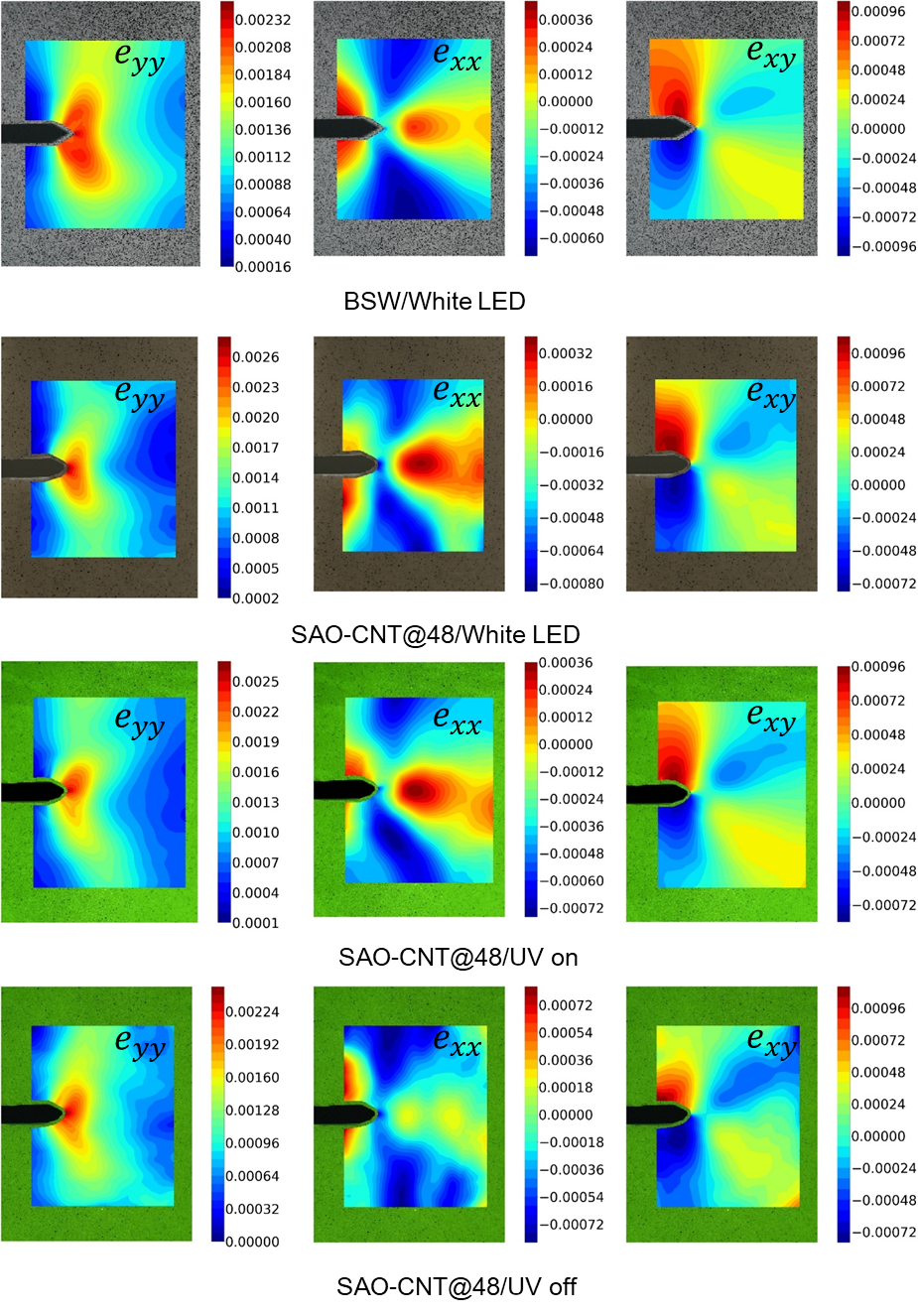


**Figure S10.** Distribution of strain component fields in the crack tip vicinity in the compact tension shear (CTS) specimens measured using BWSP and ML-DIC skins under various lighting conditions.


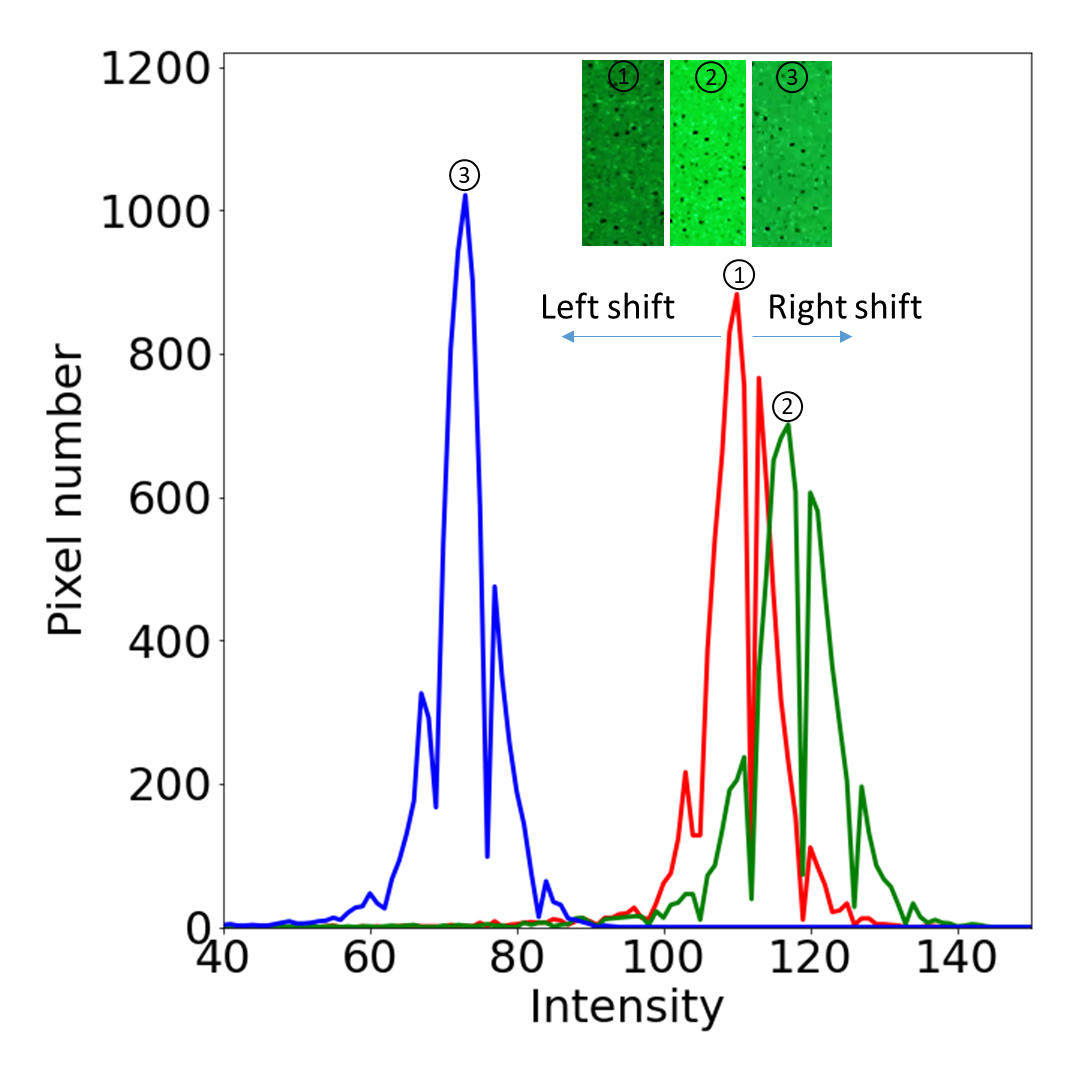


**Figure S11.** Shift in the grayscale intensity distribution to the right is attributed to an increase in ML, whereas the shift in the grayscale intensity distribution to the left is attributed to decaying PersL. The histograms correspond to the photographs shown in Figure 6(a).


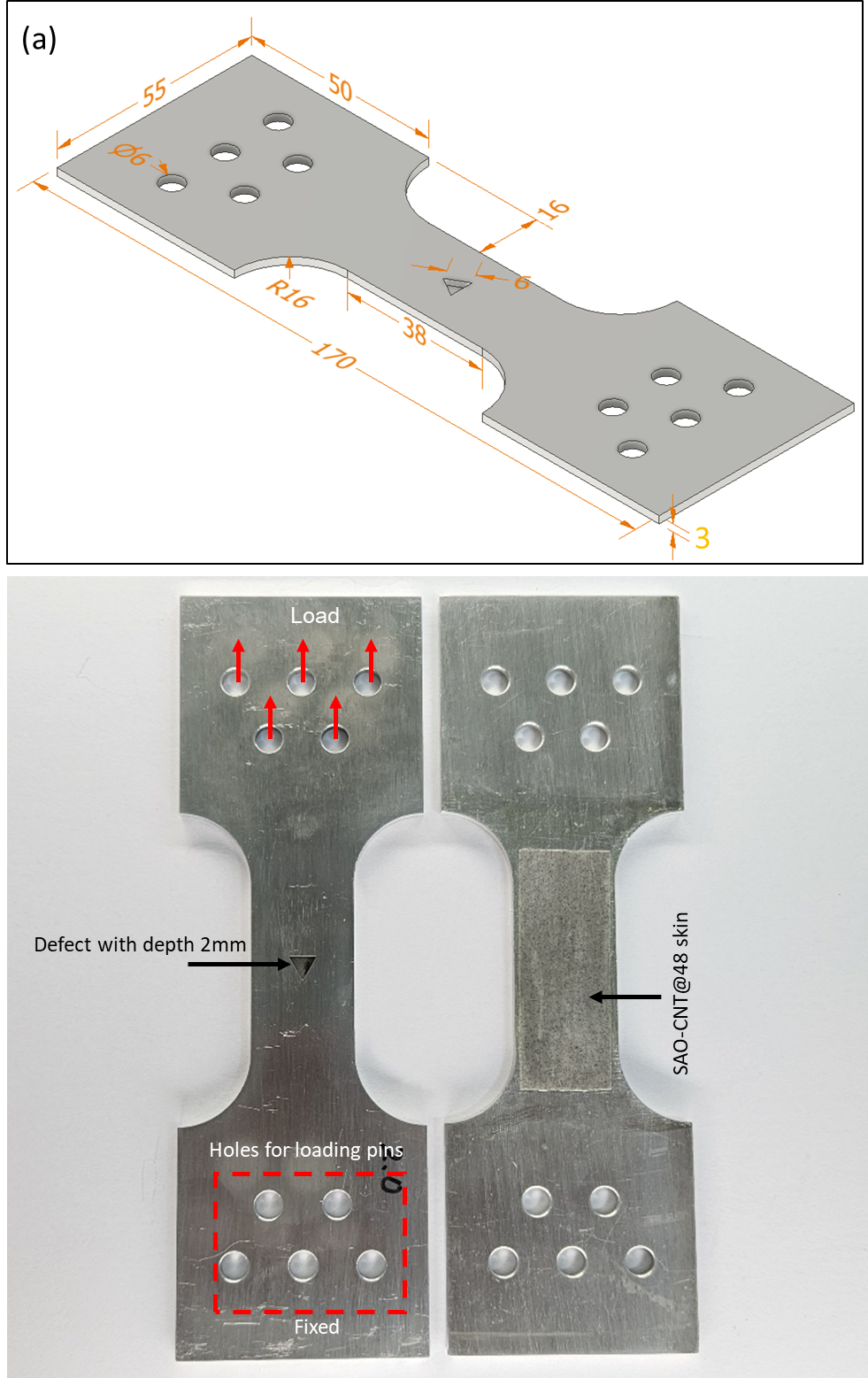


**Figure S12.** (a) Schematic of a uniaxial tension specimen with a subsurface defect of triangular geometry (b) Actual specimen made from aluminum with the affixed SAO-CNT@48 skin on the surface opposite to the defect.


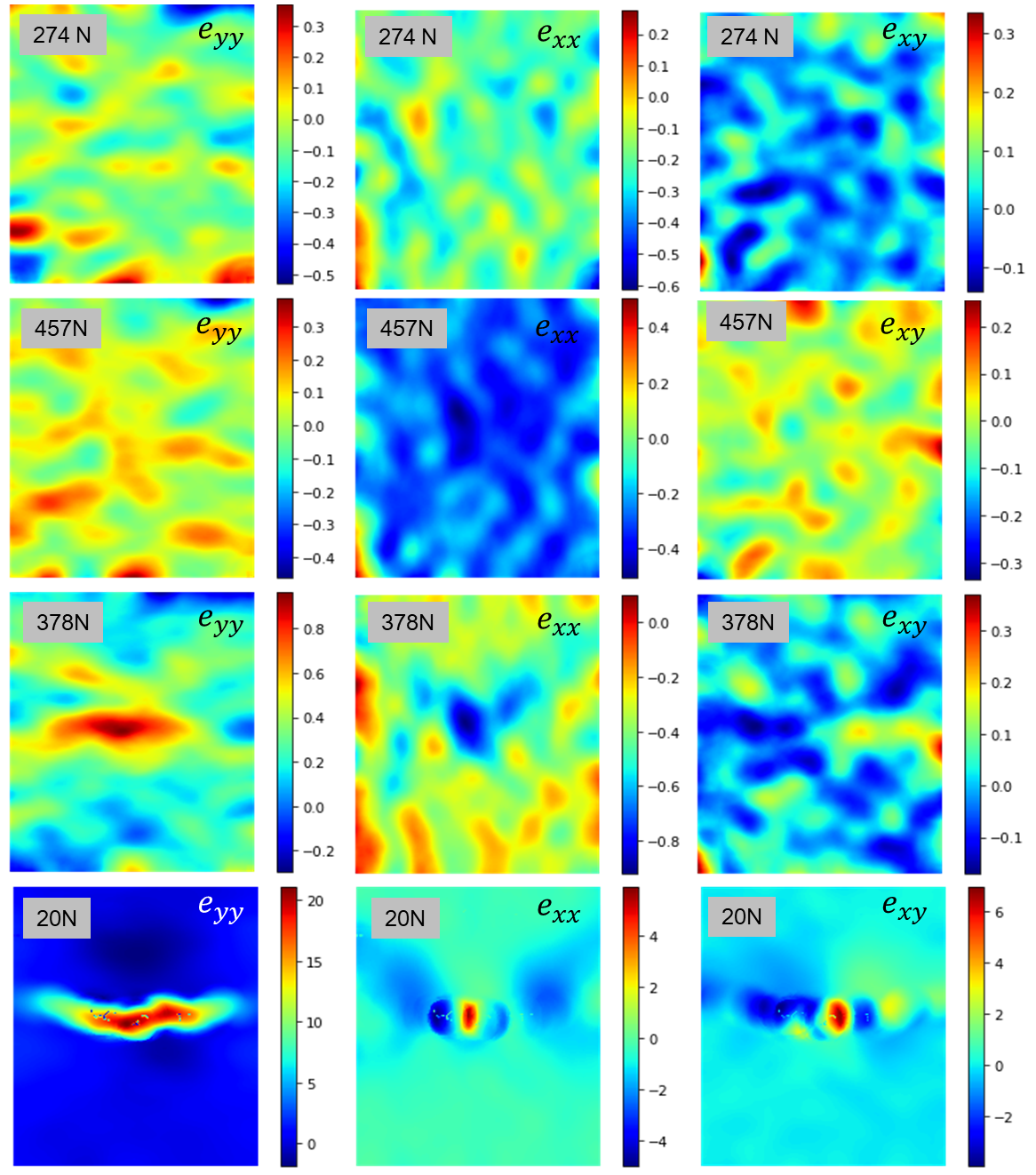


**Figure S13.** Strain component maps corresponding to the photographs shown in Figure 6(d).


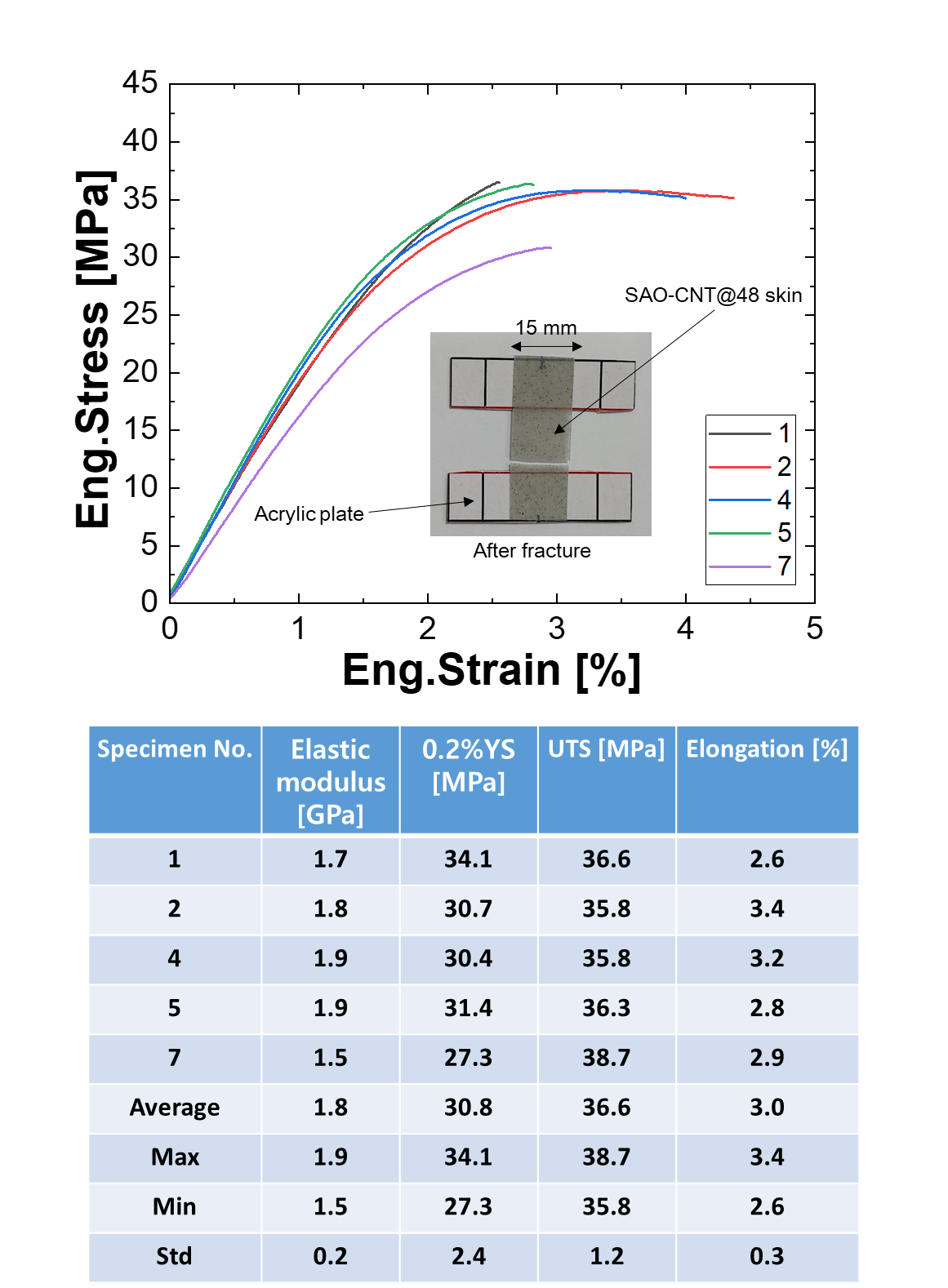


**Figure S14.** Results of the uniaxial tension test of the ML-DIC skin (SAO-CNT@48) performed to determine the fracture strength and strain. The skin was attached between acrylic plates at both ends to hold the skin during the test. The load was applied at a rate of 0.05 mm/s. The average results of seven tests are shown in the table.


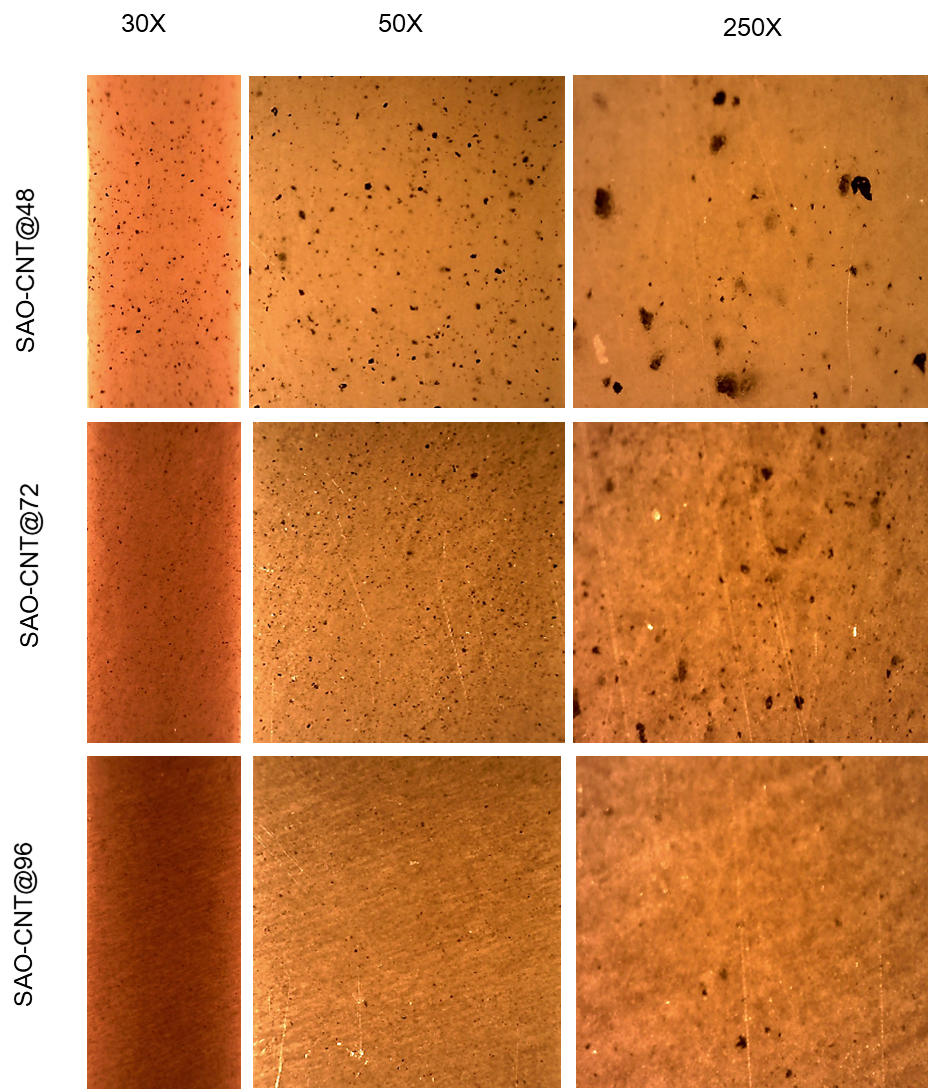


**Figure S15.** Photographs of the ML-DIC skins at magnifications of 30×, 50×, and 250× were obtained using a digital microscope.


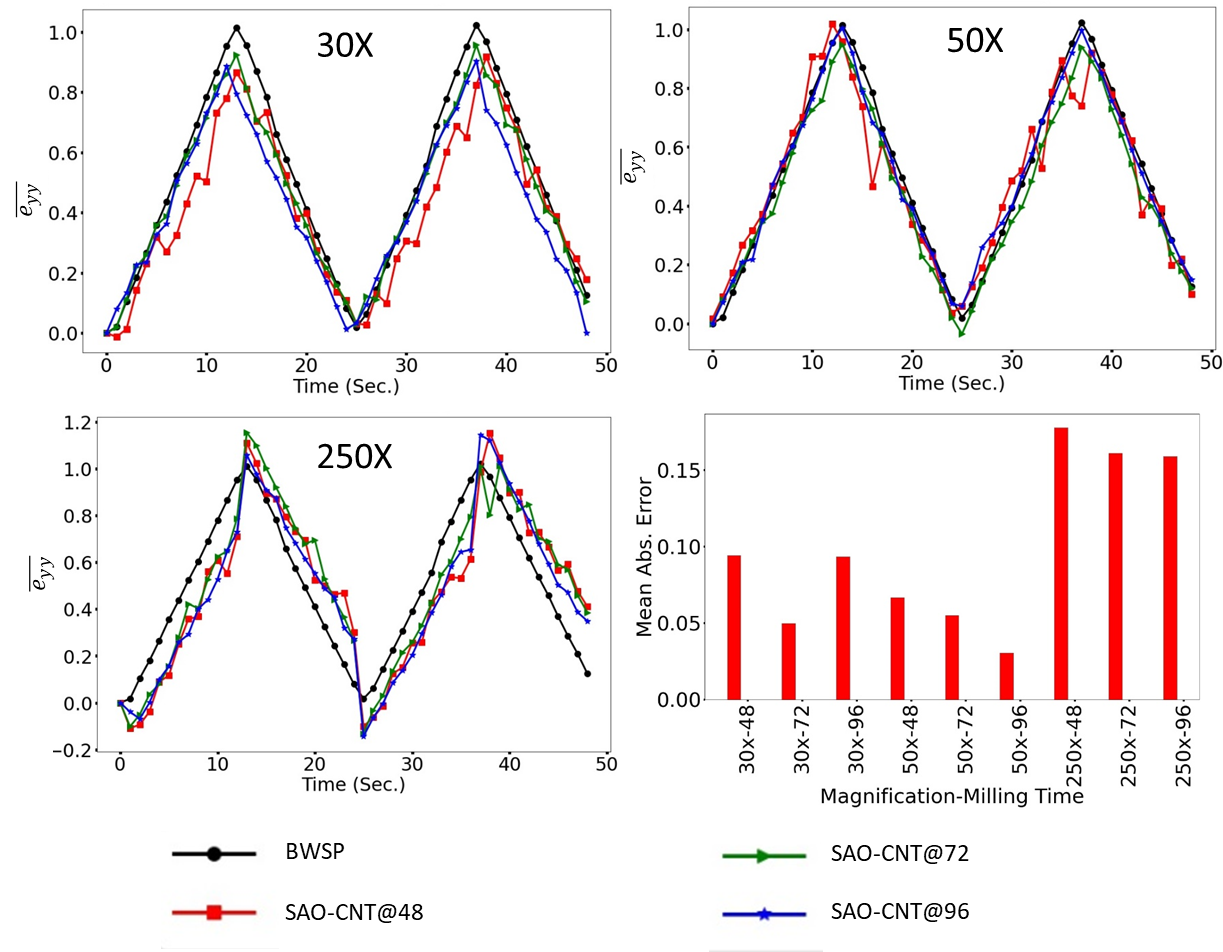


**Figure S16.** Longitudinal strain measurements at magnifications of 30×, 50×, and 250× using digital microscopic images. DIC measurements are conducted on the CNT-containing ML-DIC skins. The longitudinal strain from each ML-DIC skin is compared with that of the BWSP, which is the same as that shown in Figure 4. The MAE errors in each ML-DIC skin sample are calculated using the BWSP as a reference.


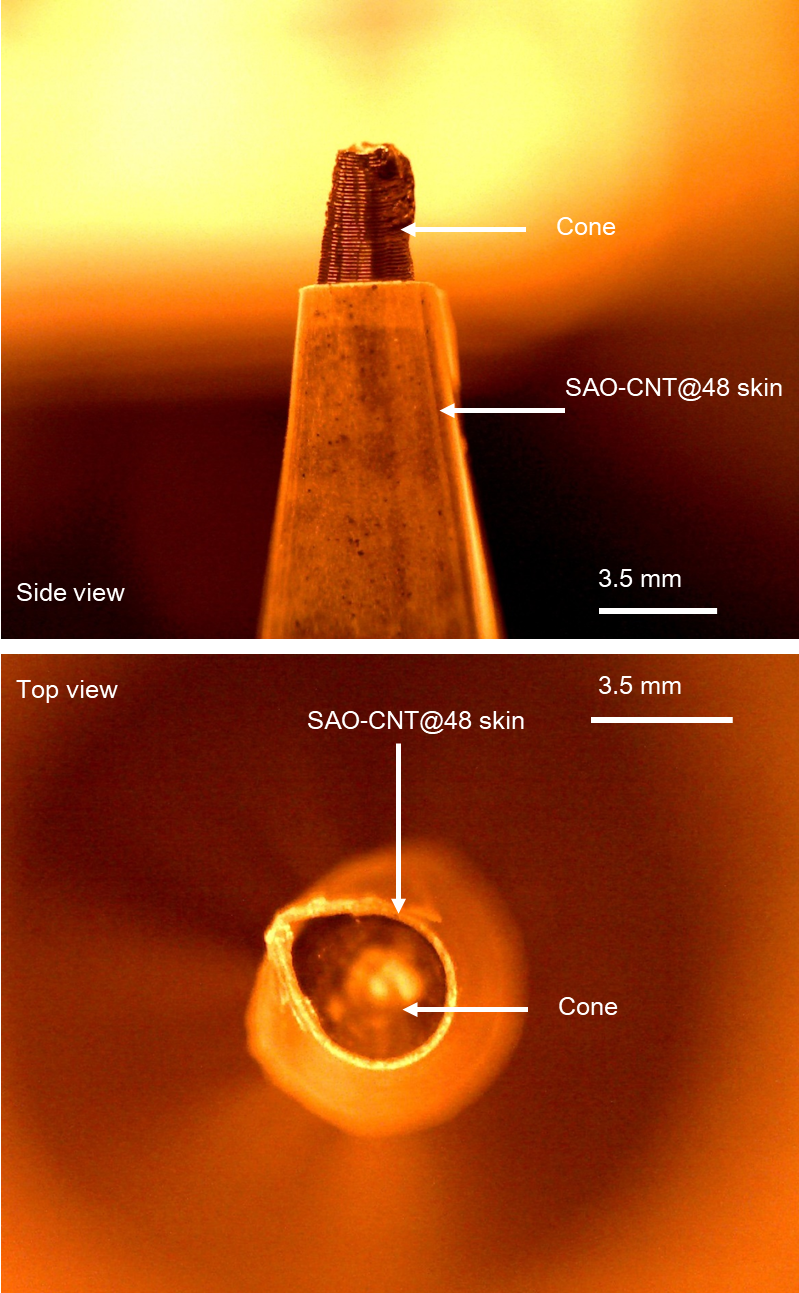
**Figure S17.** Measurement of the flexibility of DIC-ML skin (SAO-CNT@48) using an acrylic resin cone. The images were captured using digital microscopy at 30×.


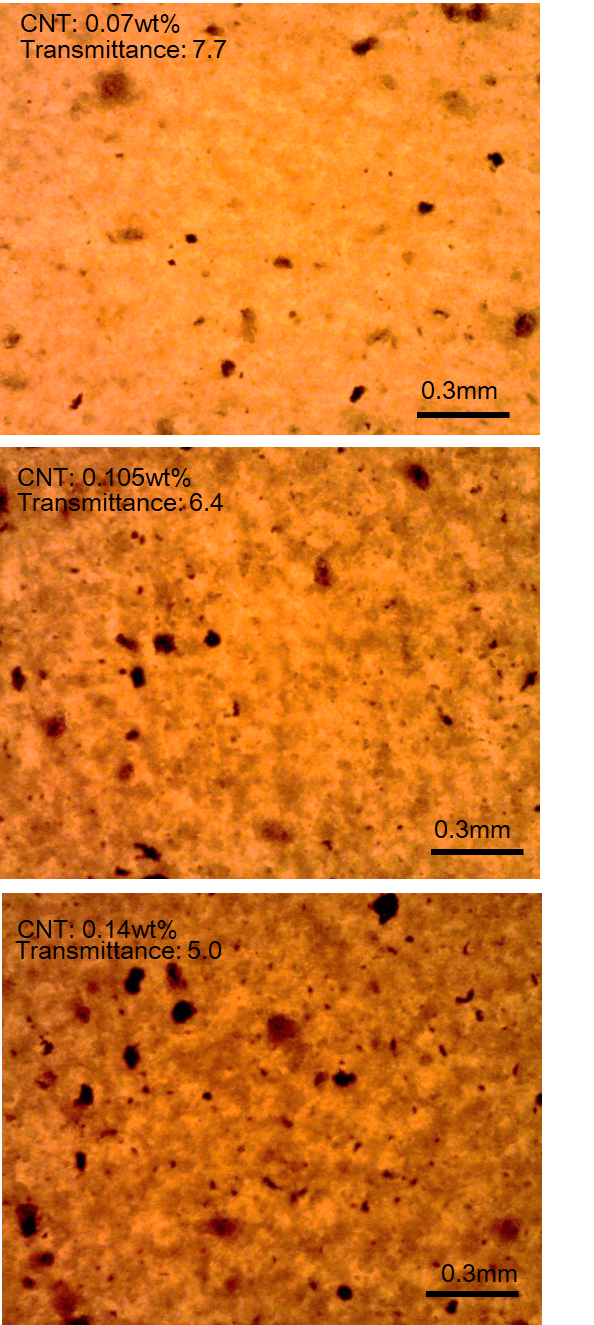


**Figure S18.** Optical images of DIC-ML skins with varying CNT wt%, where the milling time for mixing CNT, SAO, and acrylic resin was 48 h. The images were captured using digital microscopy at 250x magnification.
